# Inequality and psychological well-being in times of COVID-19: evidence from Spain

**DOI:** 10.1007/s13209-021-00255-3

**Published:** 2021-11-09

**Authors:** Monica Martinez-Bravo, Carlos Sanz

**Affiliations:** 1 CEMFI, Madrid, Spain; 2 Bank of Spain, Madrid, Spain

**Keywords:** Inequality, COVID-19, Well-being, D31, I14, J31

## Abstract

Using two novel online surveys collected in May and November 2020, we study the consequences of the COVID-19 pandemic on Spanish households. We document a large and negative effect on household income. By May 2020, the average individual lived in a household that had lost 16% of their pre-pandemic monthly income. Furthermore, this drop was highly unequal: while households in the richest quintile lost 6.8% of their income, those in the poorest quintile lost 27%. We also document that the pandemic deepened the gender-income gap: on average, women experienced a three-percentage point larger income loss than men. While this is consistent with previous findings in the literature, in this paper we document that this effect is driven by women from middle-income households with kids. Finally, we provide evidence that Spanish individuals experienced moderate declines in their levels of psychological well-being. This effect is not different for individuals living in rich or poor households, but the reasons behind well-being losses do differ: richer individuals are more concerned about loss of contact with dear ones, while low-income individuals are more likely to mention loss of income and employment as a key source of emotional distress.

## Introduction

The COVID-19 pandemic has had a profound impact on the lives of individuals throughout the world. Most countries have experienced restrictions to mobility, interpersonal contact, and economic activity that have affected income levels and the well-being of individuals. The burden of the COVID-19 shock is likely to have been different across groups of individuals. A number of studies, recently summarized by Stantcheva (2021), provide evidence that the COVID-19 pandemic is likely to deepen inequality along a number of different dimensions. Foremost among them is the potential of the COVID-19 shock to increase income inequality across individuals of most countries: loss of employment has been higher among temporary workers and low-skilled occupations have been most heavily affected by lockdowns due to the difficulties in conducting them remotely, as shown by Adams-Prassl et al. (2020).

Despite the indications that the COVID-19 may have exacerbated inequality, documenting the precise effects is difficult. Inequality is typically measured using large household surveys implemented by national statistical agencies. However, these data usually come with a substantial delay. For instance, the latest release by Eurostat of the Statistics on Income and Living Conditions (EU-SILC) dates from April 2021.^1^ However, the release only provides data until 2019. Furthermore, when the data corresponding to the year 2020 become available, they will provide a snapshot of the level of inequality, but will not uncover the dynamics across critical points in time of the pandemic. The delays in the availability of official statistics also hinder the ability of an adequate and timely policy response to the changing reality.^2^ A number of recent studies have explored alternative data sources. For instance, Chetty et al. (2020) and Aspachs et al. (2020) use data from the banking sector, while Clark et al. (2020) and Adams-Prassl et al. (2020) use data from online surveys.

In this paper, we contribute to this literature through the collection of two new online surveys representative of the population of Spain. Due to the characteristics of the Spanish economy, the country is particularly vulnerable to the economic shock of COVID-19. Spain has one of the largest shares of temporary workers in the EU, and it also has a high dependency of the tourism sector, which has been highly disrupted during the COVID-19 pandemic (Banco de España 2021). Furthermore, Spain has been severely affected by the pandemic, particularly at the initial stages, being one of the countries in the EU with the largest number of deaths per capita.^3^

We collected two online surveys during the months of June and December of 2020 from a large sample of individuals who reside in Spain. In this study, we focus on the 2678 individuals that completed both questionnaires and provided full information in key variables such as monthly income, occupation, and measures of well-being. Our resulting sample is representative of the Spanish population in terms of gender, education, and region of residence. Our sample also provides a good approximation of the pre-pandemic income distribution in Spain. Furthermore, we weight observations using sampling weights to guarantee that our findings are representative at the level of the Spanish population. We collected rich information on individuals income levels and labor conditions. In particular, we asked respondents about their levels of individual and household disposable monthly incomes at the time of each survey using narrow income brackets.^4^ We also asked them how these income levels have changed since the beginning of the pandemic. Hence, we can trace the evolution of their incomes during the most intense phase of the pandemic. In addition to this, we collected broad measures of well-being and asked individuals for the reasons behind declines in life satisfaction. These measures allow us to go beyond income measures and document how the socioeconomic situation has affected their well-being.

Using these data, we provide evidence that the COVID-19 pandemic represented a large and unequal shock for the Spanish population. We examine how the decline in income has affected households across the income distribution. We show that the poorest households experienced substantially larger shocks relative to richer households. While households in the richest quintile of the distribution had lost 7% of their household income by May 2020, the decline was of 28% for those in the poorest quintile. These effects are comparable to those experienced in the UK and the USA and larger than those in Germany (Adams-Prassl et al. 2020).^5^

Next, we study the loss of income by gender. On average, women’s incomes dropped by around three-percentage points more than men’s by May 2020, relative to their 2019 income. This difference persisted by November 2020. There are also important differences along the income distribution: while men and women living in the richest or poorest quintiles experienced a similar income loss, females living in the middle quintiles saw larger losses than males. Furthermore, while income recovered to a substantial extent for males between May and November 2020, income recovery was slower for women, particularly for those in the middle quintiles. While further investigation is needed, we provide some evidence that this result is driven by a higher propensity of women from middle-income households with kids to drop out of the labor market. This may have been motivated by the difficulties in family conciliation during the pandemic.

We then study changes in income by employment status. Our results suggest that self-employed individuals experienced the largest decreases in their disposable income. Salaried and unemployed individuals also suffered large income losses. Retired individuals experienced smaller but non-negligible losses, highlighting the strength of the pension system in accommodating negative shocks but also revealing the potential impact of the shock on other sources of income like rents. The effects were also unequal within each employment status across the income distribution, with low-income self-employed and salaried individuals experiencing larger losses than their higher-income counterparts. We also examine how the impact varied by type of contract and find that temporary workers experienced a larger drop in income, but that permanent workers also suffered large losses. We also document that the drop in income was larger among individuals in sectors considered as non-essential during the state of alarm and lockdown.

Finally, we examine the effect of the pandemic on self-reported measures of well-being. We find that individuals in the poorest quintiles had slightly lower levels of well-being relative to the richest quintiles by May 2020. Nevertheless, the income gradient of well-being is small: all quintiles report average measures between 5 and 6 (in a scale from 0 to 10). However, the reported reasons for reductions in well-being do differ substantially across the income distribution. Loss of employment or income is a more important concern for low-income individuals relative to higher-income households. In contrast, concerns over loss of contact with family members increase with income. Concern over conciliation is relatively less frequent, but it is concentrated on families in the middle of the income distribution and is particularly high among females. We also examine how the reasons for declines in well-being change between May and December. While the prevalence of feelings of uncertainty about the future declines, concerns over low contact with loved ones substantially increase across all income quintiles: 52% of respondents chose this option as a reason for loss of well-being in May; by November, the percentage increases to 74%.

Overall, these results help us uncover important dimensions of the economic effects of COVID-19 in Spanish households. First, the findings indicate that the shock was not neutral across the income distribution and exacerbated preexisting income inequalities. While previous recessions in Spain have also deepened inequality, see Bonhomme and Hospido (2017), the magnitude of the shock and the increase in inequality estimated in this paper are larger than in previous crises. This paper also shows that the patterns of recovery were different across income groups, with some groups experiencing persistent negative effects on their income. Finally, it illustrates that gender differences have differential patterns across the income distribution, with persistent negative shocks for females in middle-income households.

This paper contributes to the literature that tries to document the effects of COVID-19 on the evolution of inequality on several dimensions—see Stantcheva (2021) for a recent literature review. In the context of Spain, two previous studies have studied the evolution of inequality during the pandemic, finding mixed evidence. Clark et al. (2020) collect data from online surveys in five European countries, including Spain, between May and November 2020. This study finds small increases in inequality by May 2020 and *declines* in inequality by September 2020.^6^ However, it is unclear whether their sample is representative of the Spanish population. According to their estimates, average incomes in Spain barely change between January and May 2020 (less than 1%), and they even increase between January and September 2020. This is hard to reconcile with the large declines in GDP and with the large increases in unemployment during this period.^7^ Furthermore, their measure of income is based on respondents selecting income in large income bands. For example, their lowest income band ranges from 0 to 1250€   per month. According to EU-SILC 2019, 60% of individuals in Spain would have incomes in this interval. Hence, the data collection may hinder the ability of detect changes in the income within this large band and, hence, in income inequality.

Another approach to measure the evolution of inequality in Spain has been undertaken by Aspachs et al. (2020). They use data from a large Spanish bank on income measures and government transfers of their clients. They do find large increases in inequality during the first months of the pandemic. The Gini coefficient of pre-tax income increased from 0.45 to 0.55 between February and May 2020. Accounting for government transfers, the increase in inequality is smaller, but nevertheless positive: The post-transfer Gini coefficient increases from 0.38 to 0.42 between February and May. We differ from their study in several respects. First, their sample is limited to customers of a single bank, which may be selected toward particular types of individuals or regions. Second, we have richer information on household composition and income. Household-level income may provide a better characterization of individuals’ well-being, particularly in cases in which one member of the couple specializes in home production or for individuals enrolled in tertiary education. Third, our data allow us to measure well-being through the evaluation of psychological status, as well as other attitudinal measures.

Our paper also contributes to the literature studying emotional well-being changes after the pandemic. In this regard, our work is related to Foremny et al. (2021), who document a considerable deterioration of mental health during the pandemic in Spain. While both studies address the broad issue of well-being, their focus is more on the incidence of mental health problems, such as depression or anxiety, while our study focuses on general emotional well-being measured using a gradient from 0 to 10. Also, our data allow us to provide evidence on the reasons for well-being loss.

The rest of the paper proceeds as follows: In Sect. 2, we describe the context in which our surveys were collected and we present the data. In Sect. 3, we present our main results. In Sect. 4 we conclude.

## Context and data

The data used in this project originate from two online surveys conducted by the authors in June and December of 2020. Since respondents are likely to answer on the basis of their situation in the previous month, we refer to each wave as corresponding to the months of May and November, respectively.^8^ The surveys were conducted by YouGov, which is a well-established data analytics firm.^9^ The company has access to a large pool of individuals that have been recruited through online adds and that regularly respond to surveys on a variety of topics. Respondents accumulate points for answering surveys and they can exchange points for small gifts.

**Context** The environment in which the two survey waves were conducted was different. In May 2020, Spain had just exited from one of the strictest lockdowns in Europe. For almost two months all out-door activities were banned. Individuals could only leave their house in order to buy necessity goods or going to work. Between mid-March and the end of April, only economic activities declared as “essential” were allowed to fully operate. The sectors considered essential were health care, nursing homes, food and basic necessities stores, pharmacies, media, transportation, financial sectors, and a few others.^10^ Sectors declared non-essential had to conduct their activities online. Starting in early May, restrictions were progressively lifted and some non-essential economic activities resumed on-site. This period was referred as the de-escalation period. By the time the first wave of our survey was conducted in June 2020, restrictions were further relaxed and many economic activities, such as retail and hospitality sector, had resumed, albeit with restrictions on hours of operation, maximum capacity, and sanitary measures. The incidence of COVID cases was low (13.8 cases per 100,000 inhabitants during the last two weeks of May 2020).^11^

By the end of June 2020, Spain entered a phase labeled as “new normality”, where most economic activity had resumed. Contact-tracing was being reinforced, and containment of the virus was pursued as a policy objective. Nevertheless, COVID-19 incidence continued to increase during the second half of 2020. In October 25, the government re-instated the state of alarm and restrictions were strengthened. By the time the second wave of our study was collected in late November 2020, there were worrying concerns about a growing COVID-19 incidence. During the last two weeks of November, incidence was 275 cases per 100,000 inhabitants.^12^ There was also substantial uncertainty regarding the type of mobility and social-gathering restrictions that would be imposed during the upcoming Christmas holidays.

**Study Sample** We surveyed individuals older than 18 that reside in Spain. We collected information on the respondents’ demographic (gender, nationality, age, region of residence) and socioeconomic characteristics (employment status, occupation, income), as well as on measures of their subjective well-being. The full questionnaire can be found in Appendices C (for the first wave) and D (for the second wave). The survey also contained an experimental part, which we do not study in this paper. The experiment should not affect our results as most of the variables used in this paper were asked before the experimental section.^13^

The sampling framework of the first wave was designed to be representative of the Spanish adult population according to age, gender, region of residence, and education level.^14^ All the individuals from the first round of the survey were re-contacted in the second wave. We are particularly interested in tracking the evolution of the socioeconomic situation of individuals over this sample period. Hence, we focus our attention on the sample to the 2678 individuals that answered the complete questionnaire in both waves and that provided information regarding their income and self-reported well-being.^15^

Our resulting sample provides a good approximation to the Spanish adult population. In Table 1, we show a number of basic statistics regarding the composition of our sample when compared to the Spanish population as measured by the National Institute of Statistics (INE) in 2019. Our sample matches quite closely the gender and age distribution of the Spanish adult population. In Table 1, we also report coverage of six different geographic regions of Spain, each comprising a few Autonomous Communities.^16^ Finally, we match reasonably well the level of education of the Spanish population. We have a slightly larger representation of tertiary educated respondents and lower representation of low educated individuals. However, the disparities are moderate.^17^ In order to further strengthen the representativeness of our sample, in the rest of the analysis we use sample weights.^18^

**Summary Statistics** In Table 2, we present additional summary statistics of the main variables used in the analysis. Respondents’ ages range between 18 and 91 years old, with an average of 48 years of age. Fifty percent of respondents are female. On average, individuals in our sample have 11 years of education, which is equivalent to finishing upper secondary education.^19^

Individuals were asked about their employment status in 2019, which we classified in five categories: salaried, self-employed, retired, unemployed, or out of the labor force, which also includes students. Among the salaried and self-employed, we collected information on whether their sector was declared “essential” by the government at the beginning of the pandemic. These sectors were allowed to continue their activities, while sectors considered “non-essential” experienced a suspension of all activities that could not be done remotely until late April 2020.

**Table 1:** Representativeness

	Spanish population	Our sample
	(source: INE)	
	(1)	(2)
Female	0.52	0.49
Ages 18–24	0.08	0.05
Ages 25–34	0.14	0.15
Ages 35–44	0.19	0.22
Ages 45–54	0.19	0.21
Ages 55+	0.39	0.37
North-East region	0.21	0.20
East region	0.14	0.15
South region	0.24	0.24
Center region	0.22	0.23
North-West region	0.09	0.10
North region	0.09	0.08
Primary education or less	0.18	0.10
Secondary education	0.29	0.22
Upper secondary education	0.14	0.17
Vocational training	0.08	0.11
Tertiary education	0.31	0.40
Observations	–	2678

Column 1 shows the population averages of each characteristic in the Spanish population. The source is INE for the year 2019. Column 2 shows the average values of each characteristic in our sample

**Table 2:** Summary statistics

	Mean	Min	Max	SD	Count
*Demographic variables*					
Age	48.14	18	91	14.13	2678
Female	0.50	0	1	0.50	2678
Education (years)	11.46	5	16	3.79	2678
*Employment status variables (2019)*					
Salaried	0.51	0	1	0.50	2678
Self-employed	0.07	0	1	0.25	2678
Retired	0.15	0	1	0.36	2678
Unemployed	0.16	0	1	0.37	2678
Out of labor force	0.11	0	1	0.31	2678
Essential	0.48	0	1	0.50	1547
*Income variables*					
Household income 2019	2688	0	24,000	2019	2678
Individual income 2019	1188	0	8000	1149	2678
% Change Household income Feb–May 2020	− 15.85	− 100	100	28.51	2678
% Change Household income Feb–Nov 2020	− 11.21	− 100	100	28.73	2678
% Change Individual income Feb–May 2020	− 15.95	− 100	100	32.83	2678
% Change Individual income Feb–Nov 2020	− 11.02	− 100	100	34.97	2678
*Well-being variables*					
Well-being May 2020	5.75	0	10	2.32	2678
Well-being Nov 2020	5.35	0	10	2.28	2678
Well-being change Feb–May 2020	− 0.38	− 2	2	0.93	2678
Well-being change Feb–Nov 2020	− 0.54	− 2	2	0.94	2678
*Reason for well-being loss variables*					
*May 2020*					
Uncertainty about future	0.82	0.00	1.00	0.38	1181
Loss contact with dear ones	0.52	0.00	1.00	0.50	1181
Loss of employment	0.25	0.00	1.00	0.44	1181
Health issues	0.14	0.00	1.00	0.34	1181
Issues with conciliation	0.08	0.00	1.00	0.28	1181
*November 2020*					
Uncertainty about the future	0.78	0.00	1.00	0.42	1312
Loss contact with dear ones	0.74	0.00	1.00	0.44	1312
Loss of employment	0.30	0.00	1.00	0.46	1312
Health issues	0.17	0.00	1.00	0.38	1312
Issues with conciliation	0.06	0.00	1.00	0.25	1312

These statistics are constructed on our baseline sample using sample weights

We collected detailed data on monthly income levels both for the individual and for their household. First, we asked for their incomes before the pandemic. In particular, we asked for their net (after-tax) *total* income, including wages, earnings from professional activities, pensions, and government transfers during the average month of 2019.^20^ Individuals were asked to select an interval that includes their level of income. We take the midpoint of each interval as a proxy of their income level.^21^ In order to make comparisons across households, we define equivalent income for a four-member household formed of 2 adults and 2 children. We follow the convention used in Eurostat and other statistical agencies and assign children a weight of 0.5 when assessing their consumption demands. Hence, we divide the reported household income by the number of adult-equivalent individuals in the household, and then multiply by three, which corresponds to a household of 2 adults and 2 children. On average, the monthly disposable income of a household with four members in 2019 was of 2668€   per month. We use the same scale to elicit the level of income of the individual respondent. Our average respondent earned 1188€   per month.

Individuals were also asked about how their household and individual incomes had changed at the time of responding with respect to their income at the start of the pandemic. We also collected this information discretely by asking individual to choose between income-change intervals.^22^ Using this information, we calculate the percent change in income by dividing the reported change in levels by the 2019 income level.^23^ The average change in household income relative to 2019 is $-16$% by May 2020 and $-11$% by November 2020. The magnitudes for changes in individual income are similar. The large drop in income by May is comparable to the drop in per capita GDP as reported by national statistics. The change in GDP per capita between the fourth trimester of 2019 and the second trimester of 2020 was of $-5.9$%. The change between the last trimester of 2019 and the last trimester of 2020 was $-9.9$%.^24^ The changes in income are very similar for household and for individual income. This is an indication of the accuracy of the information reported and of the representativeness of our data of the adult population.

Finally, we recorded information on self-reported levels of well-being. In particular we asked the following question: “In a scale from 0 to 10, where 0 indicates great discomfort or depression and 10 complete happiness, how would you evaluate your level of emotional well-being?” Individuals reported an average level of well-being of 5.8 in May 2020 and of 5.3 in November 2020. Next, we asked individuals to compare their current well-being with that from before the pandemic. We asked individuals to select one of the following options, which we codified in a scale from $-2$ to 2: it has decreased a lot (= $-2$), it has slightly decreased (= $-1$), it has remained more or less the same (=0), it has slightly increased (= 1), and it has increased a lot (=2). The average value of the codified variable is $-0.38$ in May 2020 and of $-0.54$ in November 2020. These results indicate that, on average, individuals have experienced moderate decreases in well-being, and the loss in well-being became larger over time.

Finally, individuals who responded that their well-being had decreased (slightly or a lot) were asked about the main reasons for this decrease. Individuals were offered a number of potential reasons and were allowed to select more than one. On average, individuals selected 2.4 reasons in the first wave and 2.2 in the second wave. The most frequently reported reason was uncertainty about the future, which was selected by 82% of respondents in May 2020. The subsequent reasons in order of prevalence in the May survey are: reduced of contact with dear ones (52%), concerns about loss of employment (25%), health issues (14%), difficulty to conciliate work and childcare (8%).^25^ It is interesting to examine how the motives behind the decreases in well-being changed between our two waves. While uncertainty about the future seem to have slightly declined, loss of contact with dear ones increased by 18 percentage points. The prevalence of the other motives for concern changed to a lesser extent.

## Results

### Effects on income

**Inequality across the Income Distribution** In Table 2 we described how a number of key measures of income and well-being evolved, on average, during the pandemic. In this subsection we examine whether the magnitude of these changes differ across the income distribution.

We begin by classifying individuals according to quintiles of household income in 2019. For each respondent, we compute the equivalent income for a four-member household, as described in the previous section. We then divide the sample in five quintiles, each comprising approximately 20% of respondents. Quintiles are sorted from poorest to richest, and comprise individuals in the following intervals of equivalent household income per month: quintile 1 (from 0 to 1260€); quintile 2 (from 1261 to 1950€); quintile 3 (from 1951 to 2700€); quintile 4 (from 2700 to 4050€); quintile 5 (from 4050 to 24,000€).

**Fig. 1:**
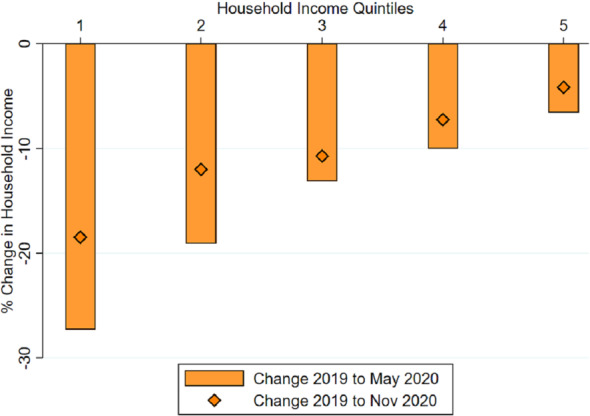
Change in household income between 2019 and 2020, by quintile. *Notes:* The bars show the percentage change in household income between 2019 and May 2020. The diamonds indicate the percentage change for the period between 2019 and November 2020. Respondents are grouped in five quintiles according to their household income in 2019: quintile 1 corresponds to the poorest quintile and quintile 5 to the richest

Figure 1 reports the effect of the COVID-19 pandemic in household income for each quintile of the income distribution. The bars represent the percentage change in household income between the pre-pandemic level (2019) and May 2020 whereas the diamonds indicate the change between the pre-pandemic level and November 2020. We find large declines in household income throughout the income distribution. The figure also reveals that the magnitude of the income decline is uneven across households: While the poorest quintile lost a 27% of their income by May 2020, the richest quintile lost around 6.5% during the same period. The income gradient of loss of income is clear, with poorest households experiencing larger percentage changes.

We also examine the extent to which incomes recovered by November 2020. Most quintiles experienced some degree of recovery. Nevertheless, the inequality-widening nature of the shock persisted: while the richest quintile had lost 4% of their income by November 2020, the poorest quintile experienced a 18% reduction.

Appendix Table 5 presents the precise statistics shown in this figure. Furthermore, it provides additional tests. In particular, we find that the differences in change of income with respect to the richest quintile are all statistically significant at the 5% level. Furthermore, the quintile dummies are jointly statistically significant at the 1% level. The rest of Tables in Appendix B provide table counterparts for each of the main figures in the text. In general, the quintile indicators and the other geographic characteristics are highly statistically significant predictors of changes in income.

In order to set our results in comparison with previous studies, we calculate the Gini coefficient in three different points in time, 2019, May of 2020 and November 2020. The resulting estimates are 0.36, 0.39, and 0.38, respectively.^26^ An increase of 0.03 points in the Gini coefficient is large in magnitude. For instance, the Gini coefficient of the distribution of income in the USA has increased by a similar amount between 1992 and 2018, which is recognized as a period of substantial deepening in the inequality of the income distribution of the USA.^27^

**Fig. 2:**
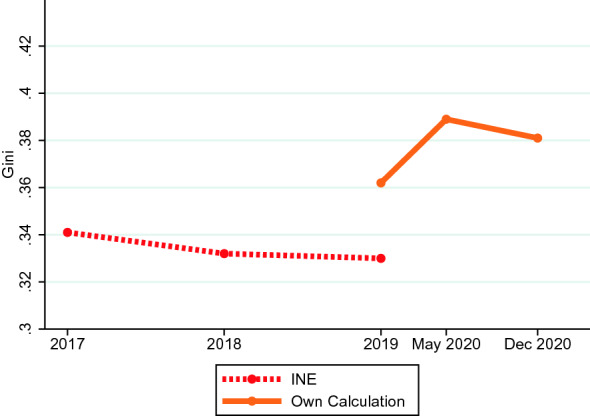
Evolution of the Gini coefficient from different sources. *Notes:* This figure shows the evolution of the Gini coefficient of disposable income as estimated from different sources. INE and our own calculations measure the Gini using household-level data

**Fig. 3:**
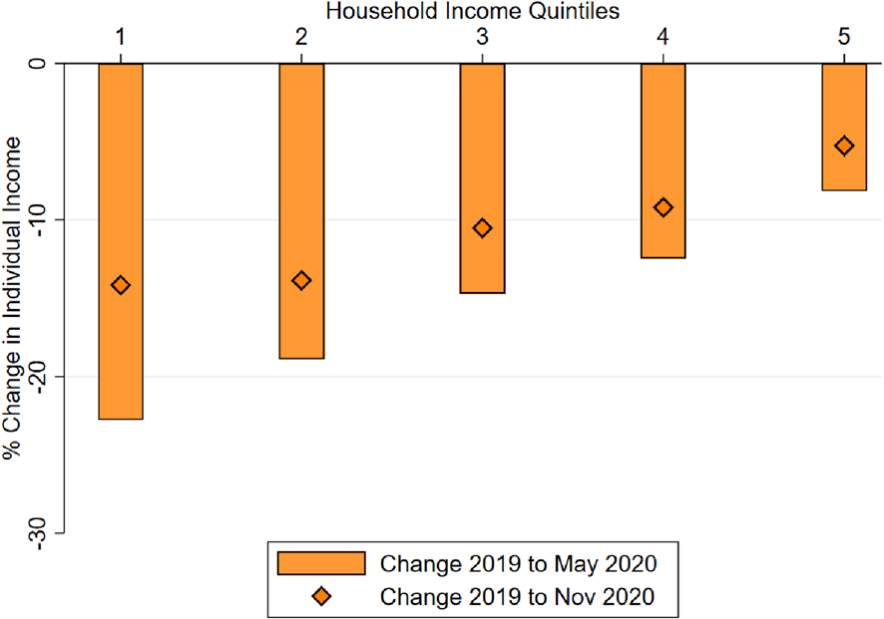
Change in individual income, by quintile. *Notes:* The bars show the percentage change in individual-level income between 2019 and May 2020. The diamonds indicate the percentage change for the period between 2019 and November 2020. Respondents are grouped in five quintiles according to their household income in 2019: quintile 1 corresponds to the poorest quintile and quintile 5 to the richest

Figure 2 plots the evolution of the Gini coefficient as obtained in our data together with the evolution from the previous years as reported by the Spanish Statistical Agency (INE). The latter source is only available until 2019. It shows a slight decline over time in the level of inequality during the period 2017–2019. Our measure of the Gini coefficient is slightly larger in magnitude, albeit in the same ballpark. More importantly, our data indicate that there has been a sizeable increase in the Gini coefficient after the outbreak of the pandemic.^28^

Next, we examine changes in individual-level income. Figure 3 presents results analogous figure to Fig.  1, when changes in income are defined at the individual-level. Note that we continue to classify individuals in quintiles according to their *household* income in 2019. Household income provides a better measure of the standards of living of individuals, particularly those out of the labor force and dedicated to home production. The results indicate that declines in individual-level income were also larger among individuals in the poorest quintiles of the household-income distribution. The income-gradient of income loss is not as large as when we examined changes in household income, but it is, nevertheless, economically significant. We also observe that there was a substantial recovery of incomes by November 2020. The recovery was similar across income deciles, hence, not altering the inequality-widening nature of the shock.

**Fig. 4:**
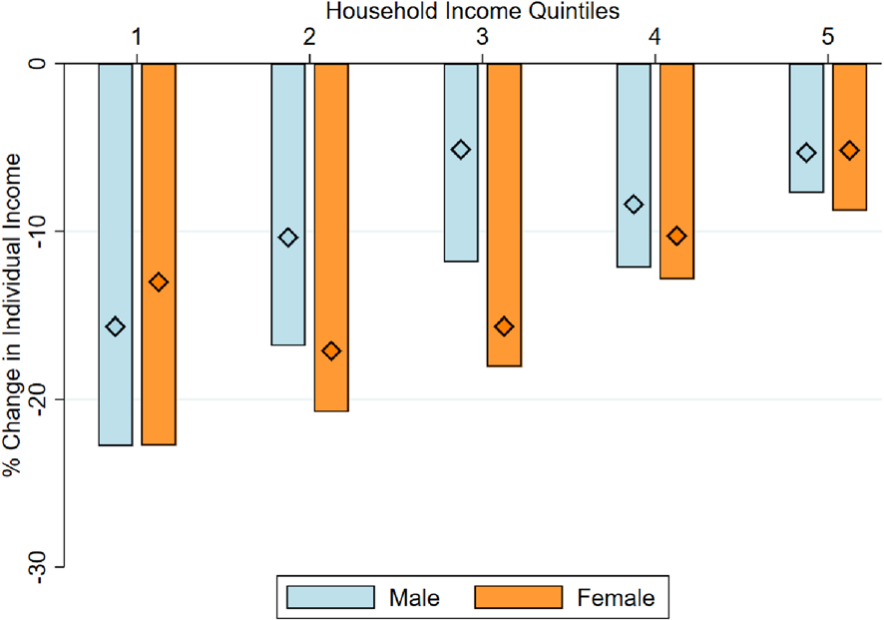
Change in individual income, by gender. *Notes:* The bars show the percentage change in individual-level income between 2019 and May 2020. The diamonds indicate the percentage change for the period between 2019 and November 2020. Respondents are grouped in five quintiles according to their household income in 2019: quintile 1 corresponds to the poorest quintile and quintile 5 to the richest. The statistics are presented by gender of the individual respondent

**Inequality across Genders** Next, we try to unbundle the economic shock on the basis of other dimensions. First, we study whether men and women have been differently affected by the shock. Figure 4 plots the change in individual income by gender, as a function of pre-pandemic household income. On average, women experienced slightly larger declines in income: a regression of the change in income on a female indicator indicates that women experienced a decline in income 3.8 percentage points larger than men by May 2020, and of 3.9 by November 2020 (both estimates are statistically significant at the 5% level). Furthermore, the analysis by income quintile uncovers interesting differences across the income distribution. The larger drop in income of women relative to men is taking place in households in the middle of the income distribution (quintiles 2 and 3, in particular). For the poorest and richest ones (quintiles 1,s 4 and 5), the gender gap in income loss is much smaller. Furthermore, while income recovered to a substantial extent for males between May and November 2020, the process of income recovery was slower for women, particularly those in the middle quintiles. One possible explanation for the larger and more persistent decline in income of middle-class women may be that some females may have been driven out of the labor-force during the pandemic, as they undertook a larger share of the responsibilities in home production during the pandemic (Farre et al. 2020).

To examine this possibility, in Appendix Fig. 13 we show the share of respondents that kept their job, were under a temporary lay-off scheme, or lost their job by May 2020. We report these statistics by gender and income group. The sample is restricted to salaried workers, which represent 51% of our sample. The results indicate that the largest gender gap in terms of loss of employment takes place for income group 2 (which aggregates quintiles 2 and 3). More generally, these results are consistent with previous studies that have pointed out that the COVID-19 pandemic has widened the gender gap. For instance, Alon et al. (2021) find that, contrary to previous recessions, the economic downturn generated by COVID-19 has generated larger employment losses for women than for men.^29^ Our results point to a similar direction for the Spanish case. Furthermore, we provide evidence that the widening of the gender gap might have been specific to women living in middle-class households.

In Appendix Table 15 we provide further suggestive evidence of the potential drivers of these effects. In particular, we present the results of regressing an indicator for having lost employment (more specifically, becoming unemployed or being under a temporary layoff scheme (ERTE)) on a female indicator, a high-income indicator, and the interaction of the two. To streamline the presentation the high-income dummy takes value 1 for quintiles 2 and above.^30^ Finally, we divide the sample in two groups: individuals with children (columns 1 and 2) and without children (columns 3 and 4). The results suggest that the higher propensity of high-income women to drop out of the labor force is present only in families with children. For instance, in column 1 we observe that, among low-income individuals with kids, females are less likely to drop out of the labor force than men (0.012 lower probability). However, among high income individuals, females are 9 percentage points more likely to drop out of the labor force than men (0.09 = − 0.012 + 0.102). When we compare this result with column 3 (no kids) we find that this effect disappears: high income women without kids are not more likely to drop out of the labor force than men of similar income. Columns 2 and 3 show that the results are robust to including dummies for types of occupation interacted with a female dummy. If anything the results become stronger, with the interaction term of column 2 increasing in magnitude and significance (the *p*-value is 0.137). This evidence suggests that women across the income distribution experienced a different evolution of their labor force participation, particularly when they had kids. In particular, it suggests that family conciliation difficulties may have been an important determinant of dropping out of the labor force. A key question is why these conciliation difficulties may not have been at play for women with kids in the poorest quintile. One potential answer is that for these women, dropping out of the labor force may not have been an option because their earnings may have be needed to sustain the family.

**Fig. 5:**
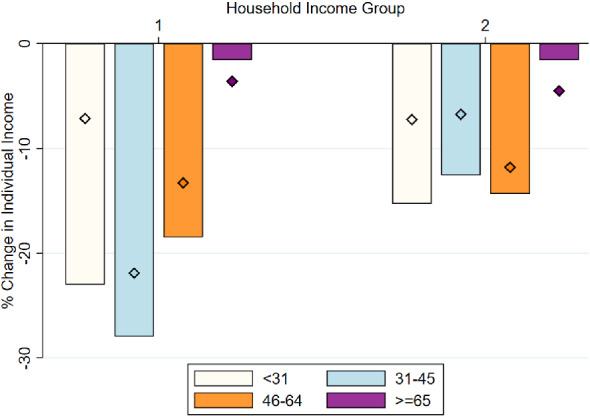
Change in individual income, by age. *Notes:* The bars show the percentage change in individual-level income between 2019 and May 2020. The diamonds indicate the percentage change for the period between 2019 and November 2020. Respondents are grouped in two income groups: group 1 includes individuals in the poorest two quintiles, whose equivalent household incomes were lower than 1950€   per month in 2019; group 2 includes individuals from the top three quintiles. We decompose individuals by age groups as shown in the legend

**Inequality across the Age Distribution** In Fig. 5, we examine differential effects by age groups. We decompose the population in four age groups: younger than 31, 31 to 45, 46 to 64, and over 65 years of age. Note that due to sample size limitations we bundle the 5 quintiles in just two income groups. Group 1 includes individuals from the poorest two quintiles, whose equivalent household income is below 1950€   per month in 2019. Group 2 includes individuals from the top 3 quintiles. With the only exception of individuals above 65 years of age, all other age groups experienced large declines in individual income. This finding illustrates the strength of the pension system in shielding households from negative macro-economic shocks. Consistent with previous findings, individuals that belong to poorer families experience larger declines in income relative to individuals from richer families. Interestingly, among individuals younger than 65 and once we hold constant the income group, there is not a clear relationship between age of the individual and the magnitude of the initial income shock experienced by May 2020. In other words, the heterogeneity in the magnitude of the shock is larger across the income distribution than across the age distribution. Nevertheless, the speed of recovery does seem to differ by age, with individuals below 31 years of age recovering faster relative to older ones. The persistence of the initial income shock seems particularly larger for individuals with ages between 31 and 64 in the poorest group.

**Fig. 6:**
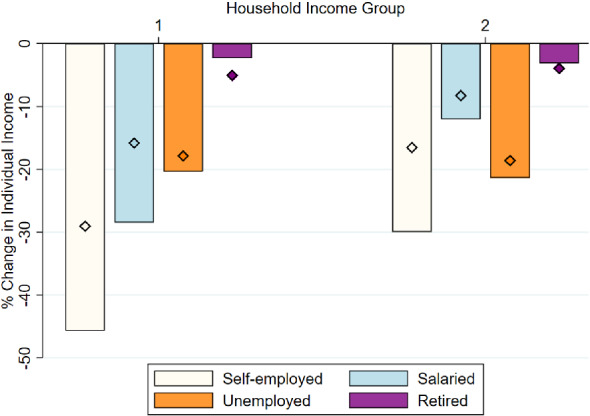
Change in individual income, by employment status. *Notes:* The bars show the percentage change in individual-level income between 2019 and May 2020. The diamonds indicate the percentage change for the period between 2019 and November 2020. Respondents are grouped in two groups: group 1 includes individuals in the poorest two quintiles, whose equivalent household incomes were lower than 1950€   per month in 2019; group 2 includes individuals from the top three quintiles. We decompose individuals by the employment status they had before the pandemic, in 2019, as shown in the legend

**Inequality across Pre-pandemic Employment Status** Next, we examine the change in incomes of individuals by their employment status before the pandemic. We group individuals in one of the following categories: self-employed, salaried worker, unemployed, and retired, according to their status shortly before the pandemic. We continue to group individuals in two different income categories as described in the previous figure. Figure 6 shows the results. Self-employed individuals are the ones that experience the largest decline in income in both income categories, particularly in May 2020. Salaried individuals also experience large drops in income, particularly among the poorest income group. The unemployed group experiences similar declines in income across both groups.^31^

Finally, retired individuals experience modest declines in their incomes. While self-employed and salaried individuals experience the largest drops in income, they also exhibit the largest recoveries by November 2020. Nevertheless, even by November 2020 these individuals had lost between 10 and 30% of their pre-COVID-19 income. These results indicate that the nature of the economic activity of individuals was a key determinant of the intensity of the economic shock generated by the COVID-19 pandemic.

**Fig. 7:**
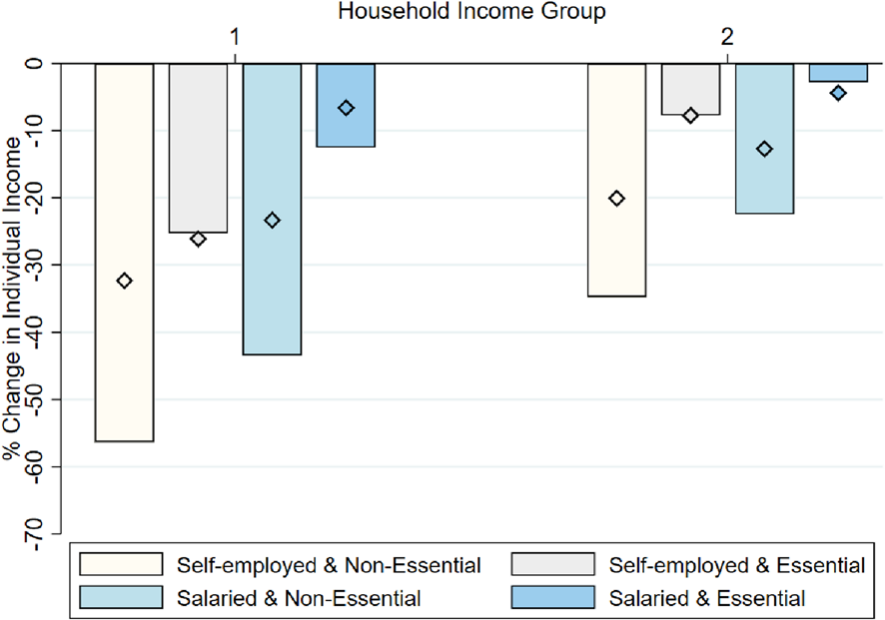
Change in individual income of salaried and self-employed income. *Notes:* The bars show the percentage change in individual-level income between 2019 and May 2020. The diamonds indicate the percentage change for the period between 2019 and November 2020. Respondents are grouped in two income groups: group 1 includes individuals in the poorest two quintiles, whose equivalent household incomes were lower than 1950€   per month in 2019; group 2 includes individuals from the top three quintiles. The sample is restricted to individuals who were salaried workers or self-employed in 2019. We decompose individuals in both sectors by whether their sector was declared *essential* or not during the state of alarm declared in March 14, 2020

In Fig. 7 we focus on self-employed and salaried individuals. Among this set of respondents, we collected information on their sector of activity. In particular, we asked them whether their type of economic activity was declared as “essential” during the state of alarm declared in March 14, 2020.^32^ The figure indicates that individuals in activities declared as non-essential were hit harder by the economic shock. However, even individuals in essential sectors experienced large declines in their incomes. By November 2020, the self-employed in non-essential sectors experienced the largest recovery, while those in essential sectors had more persistent shocks. These results highlight that even individuals in essential sectors that were allowed to continue their operations experience significant negative spillovers in their overall economic activity.

**Fig. 8:**
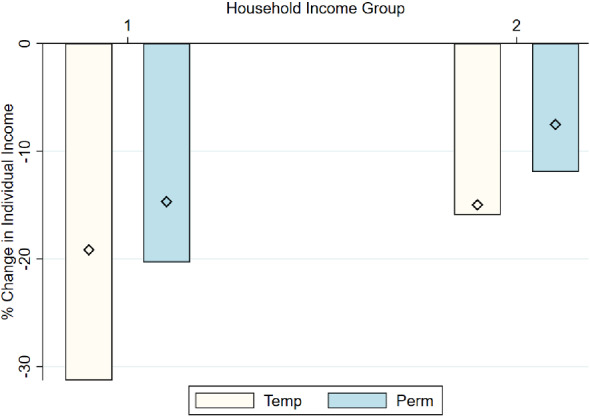
Change in individual income of salaried employees, by type of contract. *Notes:* The bars show the percentage change in individual-level income between 2019 and May 2020. The diamonds indicate the percentage change for the period between 2019 and November 2020. Respondents are grouped in two income groups: group 1 includes individuals in the poorest two quintiles, whose equivalent household incomes were lower than 1950€   per month in 2019; group 2 includes individuals from the top three quintiles. The sample is restricted to individuals who were salaried workers in 2019. We decompose individuals depending on their job status by 2019. In particular by whether they had a temporary or permanent contract

Finally, we study differences by type of contract: temporary or permanent. Spain is characterized by a “dual” labor market, with a very high share of temporary workers. This has been shown to have an impact on how the labor market reacts to recessions, generating large employment losses, especially among temporary workers (Bentolila et al. 2012). In Fig. 8 we plot income change among salaried workers, divided by the type of contract they had in 2019. The figure indicates that income losses have been higher for temporary workers, but also substantial for permanent ones. However, the speed of income recovery seemed larger for temporary workers than for permanent ones.

**Inequality and Changes in Employment Status during the Pandemic** One of the key predictors of loss in income is becoming unemployed or transitioning to a temporary layoff scheme (i.e., ERTE). The indicators of these rough categories of changes in job status can explain 33% of the variation in changes in individual-level income. However, the incidence of job loss is strongly associated with income quintile. Appendix Fig.  13 already documents this pattern: individuals in the bottom 20% of the income distribution are at least twice as likely to lose their job or transition to ERTE than individuals at higher levels of income.

**Fig. 9:**
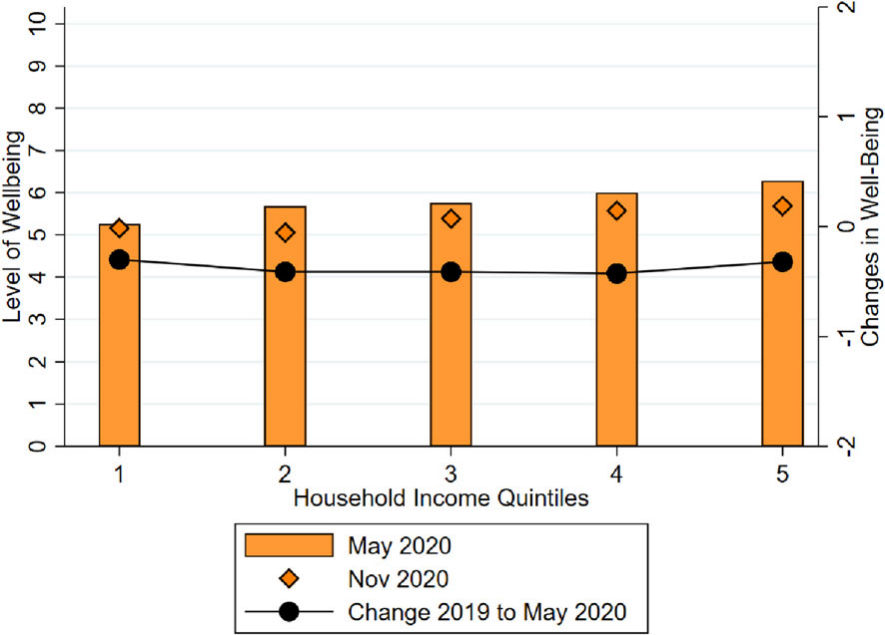
Well-being levels and change, by income. *Notes:* The bars show the level of well-being by May 2020 in a scale from 0 to 10. The diamonds indicate the level of well-being by November 2020. Respondents are grouped in five quintiles according to their household income in 2019: quintile 1 corresponds to the poorest quintile and quintile 5 to the richest. The dots correspond to the changes in well-being between the start of the pandemic and May 2020. The categorical change has been coded on a scale from $-2$ to 2, which is represented in the right-hand-side y-axis. Positive levels correspond to improvements in well-being, while negative levels represent declines in well-being

### Effects on well-being

Evidence from other countries suggests that the pandemic has had a negative impact on psychological well-being (Ettman et al. 2020). Here we provide novel evidence for the Spanish case. Figure 9 plots the reported (individual) well-being on a 0–10 scale for individuals across 2019 household income quintiles. We find that individuals in the poorest quintiles have slightly lower levels of well-being relative to the richest quintiles by May 2020. Nevertheless, the income gradient of well-being is small: all quintiles reporting average measures between 5 and 6.2. The levels of well-being by November 2020 is slightly lower for most quintiles. We also asked individuals how their subjective well-being has changed with respect to the pre-pandemic level and we codified this measure between $-2$ and 2, where positive values mean improvements in well-being and negative values mean declines. We plot reported changes in well-being as dots whose values can be found in the right-hand side y-scale. On average, people report having experienced moderate decreases in well-being, with an average value of our codified variable of $-0.38$. However, there is not a clear income gradient in the change in well-being. Overall, these results suggest that there are small differences in levels or changes in aggregate well-being across the income distribution. If anything, low-income individuals have lower levels of well-being, but this is something that seems to pre-date the COVID-19 pandemic.

**Fig. 10:**
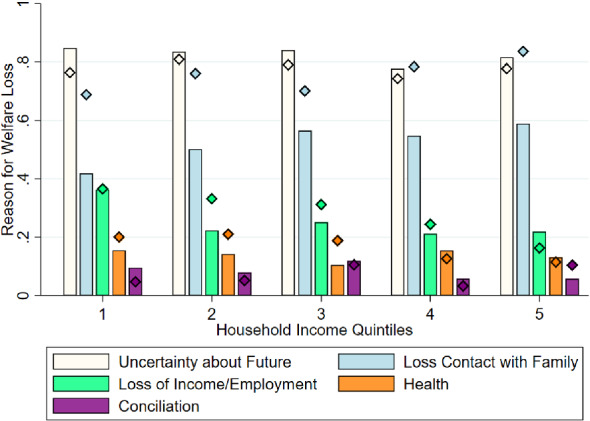
Reasons for loss of well-being, by income. *Notes:* The bars indicate the fraction of respondents that selected each reason for the decline in well-being in May 2020. The diamonds indicate the fraction of respondents that selected each reason by November 2020. The questions were only asked to respondents that indicated that the experienced a decline in their levels of well-being. Individuals could select multiple reasons

Nevertheless, there are important differences along the income distribution on the *factors* that influence emotional well-being. We illustrate this in Fig. 10. Among individuals who responded that their well-being had decreased (slightly or a lot) during the pandemic, we asked for the main reasons. Individuals were offered a number of potential reasons and were allowed to select more than one. In the figure, we report the fraction of individuals from each quintile that select a given category. We focus on the 5 most prevalent reasons: uncertainty about the future, reduced of contact with dear ones, worries about employment loss, health issues, and difficulties in conciliation of work and childcare.

The figure reveals at least three important findings. First, uncertainty about the future is the reason that is most frequently mentioned (by around 80% of individuals). Interestingly, this concern only decreases mildly by November 2020, which suggests that households perceived uncertainty remained high at that point. Second, we find a clear income gradient for the following two main concerns: while concerns over loss of employment was a more prevalent concern among poorer deciles, the opposite happens for worries over loss of contact with dear ones. The other reasons do not have a clear income gradient. Finally, there were some sizable changes over time. The most salient one refers to loss of contact with dear ones, which increase by 20 points in the prevalence of a main concern (from 52% in May 2020 to 72% in November 2020). The increase is similar across all income deciles. This highlights that the psychological costs of restriction to inter-personal contact may have been sizable during this period.

## Conclusion

In this paper, we have presented novel evidence on the consequences of the COVID-19 pandemic on Spanish households. The data used in this paper come from two online surveys collected during May and November of 2020. The use of online surveys provides a powerful tool to examine, almost in real time, the evolution of household incomes during one of the worst economic crises of the last decades, as well as their consequences on psychological well-being.

The main findings of this study are the following. First, we document a large and negative effect on household income. By May 2020, the average household in Spain had lost 16% of their pre-pandemic income. By November 2020, the average household had only recovered 5 points of this drop. Second, the size of the economic shock was highly unequal. While households on the richest quintile lost 6.8% of their income by May 2020, the drop in income by the poorest quintile was 27%. As a result of this shock, the Gini coefficient experienced an increase of 3 points, from 36 in 2019 to 39 by May 2020. This increase is comparable to the cumulative increase in the Gini coefficient of the income distribution of the USA in the last three decades, which is a well-known case of large increase in inequality (Piketty et al. 2018). Third, the negative effects on income were larger for women than for men. Hence, we confirm previous findings in the literature that indicated the widening of the gender gap during the COVID-19 economic crisis. In this study, we furthermore show that this is driven particularly by the income process of women living in households with middle-income levels and that have kids. These women may have experienced stronger difficulties with family conciliation but at the same time may have been more able to reduce their labor force participation relative to poorer women. Fourth, we find very large income losses for the self-employed. By May 2020, the poorest 40% of self-employed individuals had lost on average 46% of their income. While they recovered 17 points of this drop by November 2020, the magnitude of the income reduction by November 2020 is still sizable. Fifth, salaried workers also experienced large losses on their income. By May 2020, the poorest 40% of workers experienced 28% declines in income. Sixth, we find moderate declines in psychological well-being that are uniform across the income distribution. However, the reasons for loss of well-being are different across deciles: while richer individuals are more concerned about loss of contact with dear ones, the poor are more concerned about loss of income and employment as an important source of distress.

Overall, this study illustrates the importance of having access to detailed data on households finances that can be collected and processed in a timely manner. Furthermore, in contrast to other studies in the Spanish context, it defines households as the unit of analysis. Household income is likely a better measure of standards of living of a large fraction of the population, particularly in settings where some household members specializes in home production. This study also contributes to the literature by documenting the psychological effects of the crisis generated by the COVID-19 pandemic. The use of online surveys allows researchers to collect these additional metrics of well-being which are not available in administrative data or surveys from official sources.

## Figures and Tables

**Fig. 11:**
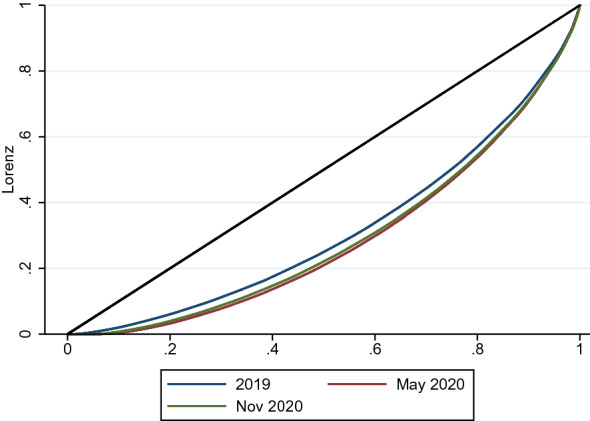
Lorenz curve of the household income distribution. *Notes:* This figure shows the Lorenz curves of the distribution of household income in three different points in time. The curve plots the proportion of overall income that earned by the to the poorest x% households

**Fig. 12:**
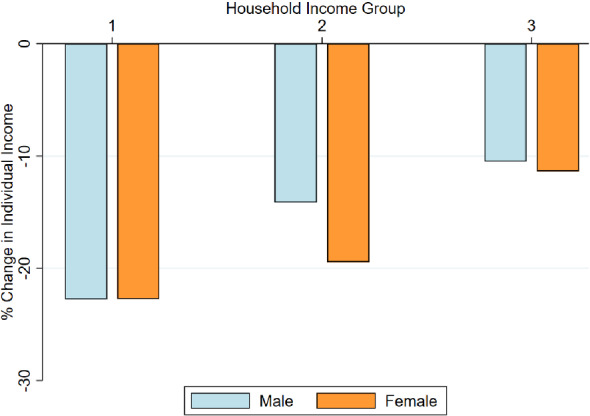
Individual income change by gender, three income groups

**Fig. 13:**
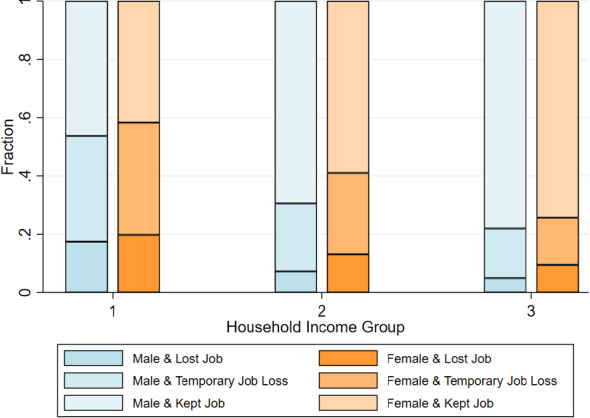
Incidence of job loss by gender (May 2020). *Notes:* This figure shows the fraction of respondents by job status in May 2020 by gender and income group. Group 1 contains the poorest quintiles, group 2 contains quintiles 2 and 3 and group 3 contains quintiles 4 and 5. The sample is restricted to salaried workers

## A Appendix Figures

See Figs. 11, 12 and 13.

## B Appendix Tables

See Tables 3, 4, 5, 6, 7, 8, 9, 10, 11, 12, 13, 14 and 15.

**Table 3:** Representativeness with respect to EU-SILC

	EU-SILC Spain 2019	Our Survey 2019
	(1)	(2)
*A. Inequality measures*		
Gini coefficient	33	36
Standard deviation	7604	7406
Percentile 50/10	2.40	2.34
Percentile 90/50	1.99	2.53
*B. Top cutoff point of each income decile*		
(*Annual income per equivalent adult*)		
First decile	6267	4620
Second decile	8847	6067
Third decile	10,815	7560
Fourth decile	12,997	9240
Fifth decile	15,015	10,800
Sixth decile	17,422	13,125
Seventh decile	20,358	15,000
Eighth decile	24,104	21,000
Ninth decile	29,907	27,300

Column 1 presents a number of inequality statistics for the Spanish population in 2019, as reported by Eurostat (EU-SILC). Column 2 reports comparable statistics estimated in our sample. In particular, both data sources are constructed using measures of household disposable income divided by the number of equivalent adults in the household

**Table 4:** Measures of inequality

Year	Gini	A(0.5)	A(1)	Variance log income	75/25	90/10	MLD	THEIL
	(1)	(2)	(3)	(4)	(5)	(6)	(7)	(8)
2019	0.362	0.107	0.208	0.501	2.500	5.833	0.233	0.223
May 2020	0.389	0.125	0.247	0.647	2.700	6.875	0.283	0.256
Nov 2020	0.381	0.119	0.232	0.574	2.500	6.900	0.263	0.249

This table provides a number of inequality statistics for household income

**Table 5:** Change in household income with respect to 2019

	Mean	Min	Max	SD	Count	Diff	se (diff)
	(1)	(2)	(3)	(4)	(5)	(6)	(7)
*Panel A. May 2020 wave*							
Quintile 1	-27.29	-100.00	100.00	39.77	541	− 20.73	2.25
Quintile 2	-19.10	-100.00	95.24	29.99	542	− 12.53	1.95
Quintile 3	-13.11	-100.00	100.00	24.32	607	− 6.54	1.44
Quintile 4	-10.01	-100.00	44.44	19.23	610	− 3.44	1.26
Quintile 5	-6.57	-76.92	3.75	13.14	378	–	–
*Panel B. Nov 2020 wave*							
Quintile 1	-18.48	-100.00	100.00	39.12	627	− 14.33	2.16
Quintile 2	-11.99	-100.00	95.24	28.84	462	− 7.84	1.71
Quintile 3	-10.72	-100.00	95.24	25.96	592	− 6.57	1.67
Quintile 4	-7.24	-100.00	95.24	22.45	591	− 3.09	1.51
Quintile 5	-4.15	-76.92	60.61	11.65	406	–	–

Columns 1 to 5 of this table present the statistics used in Fig. 1. Columns 6 and 7 examine whether the change in household income is statistically different across quintiles. We regress the change in household income on quintile dummies, omitting quintile 5. Column 6 reports the point estimates and column 7 reports the standard errors. All differences are statistically significant at the 5% level. The F-statistic of joint significance of all quintile dummies is 28.63 in panel A and 16.19 in panel B

**Table 6:** Change in individual income, by quintile

	Mean	Min	Max	SD	Count	Diff	SE (diff)
	(1)	(2)	(3)	(4)	(5)	(6)	(7)
*Panel A. May 2020 wave*							
Quintile 1	-22.76	-100.00	100.00	40.93	541	− 14.58	2.60
Quintile 2	-18.88	-100.00	100.00	34.70	542	− 10.70	2.35
Quintile 3	-14.71	-100.00	100.00	31.36	607	− 6.54	2.02
Quintile 4	-12.46	-100.00	74.07	27.85	610	− 4.29	1.99
Quintile 5	-8.17	-100.00	15.38	20.39	378	–	–
*Panel B. Nov 2020 wave*							
Quintile 1	-14.17	-100.00	100.00	41.54	627	− 8.91	2.55
Quintile 2	-13.87	-100.00	100.00	36.01	462	− 8.61	2.44
Quintile 3	-10.52	-100.00	100.00	35.26	592	− 5.26	2.48
Quintile 4	-9.21	-100.00	100.00	31.85	591	− 3.95	2.22
Quintile 5	-5.26	-100.00	74.07	21.18	406	–	–

Columns 1 to 5 of this table present the statistics used in Fig. 3. Columns 6 and 7 examine whether the change in individual income is statistically different across quintiles. We regress the change in individual income on quintile dummies, omitting quintile 5. Column 6 reports the point estimates and column 7 reports the standard errors. All differences are statistically significant at the 5% level, except for the difference between quintile 4 and 5 in November 2020, which is significant at the 10% level. The F-statistic of joint significance of all quintile dummies is 10.64 in panel A and 5.12 in panel B

**Table 7:** Change in individual income, by gender

	Mean	Min	Max	SD	Count	Diff	se (diff)
	(1)	(2)	(3)	(4)	(5)	(6)	(7)
Panel A. May 2020 wave							
Male							
Quintile 1	-22.78	-100.00	100.00	40.87	222	− 15.05	3.74
Quintile 2	-16.81	-100.00	100.00	31.64	251	− 9.09	3.19
Quintile 3	-11.85	-100.00	100.00	28.50	330	− 4.12	2.40
Quintile 4	-12.17	-100.00	40.00	26.60	345	− 4.44	2.58
Quintile 5	-7.73	-100.00	15.38	18.83	222	–	–
Female							
Quintile 1	-22.74	-100.00	100.00	41.02	319	− 13.97	3.65
Quintile 2	-20.75	-100.00	100.00	37.22	291	− 11.97	3.49
Quintile 3	-18.04	-100.00	100.00	34.14	277	− 9.27	3.35
Quintile 4	-12.85	-100.00	74.07	29.45	265	− 4.08	3.15
Quintile 5	-8.78	-100.00	0.00	22.39	156	–	–
Panel B. Nov 2020 wave							
Male							
Quintile 1	-15.68	-100.00	100.00	41.71	283	− 10.35	3.82
Quintile 2	-10.37	-100.00	100.00	32.25	220	− 5.04	2.97
Quintile 3	-5.13	-100.00	100.00	33.56	297	0.20	3.48
Quintile 4	-8.40	-100.00	95.24	31.61	329	− 3.08	3.13
Quintile 5	-5.32	-100.00	74.07	21.81	241	–	–
Female							
Quintile 1	-13.03	-100.00	100.00	41.44	344	− 7.85	3.40
Quintile 2	-17.11	-100.00	100.00	38.95	242	− 11.93	3.72
Quintile 3	-15.66	-100.00	100.00	36.13	295	− 10.48	3.36
Quintile 4	-10.29	-100.00	100.00	32.19	262	− 5.11	3.06
Quintile 5	-5.18	-100.00	74.07	20.37	165	–	–

Columns 1 to 5 of this table present the statistics used in Fig. 4. Columns 6 and 7 examine whether the change in individual income is statistically different across quintiles. We regress the change in individual income on quintile dummies, omitting quintile 5. The regression is estimated on the sample corresponding to each sub-panel. Column 6 reports the point estimates and column 7 reports the standard errors. Most differences are statistically significant at the 5% level, with the exception of quintile 4, which is not always significantly different from quintile 5. The F-statistic of joint significance of all quintile dummies and their interactions with the female dummy is 5.23 in panel A and 3.2 in panel B

**Table 8:** Change in individual income, by age

	Mean	Min	Max	SD	Count	Diff	se (diff)
	(1)	(2)	(3)	(4)	(5)	(6)	(7)
Panel A. May 2020 wave							
Age< 31							
Household income group 1	-23.01	-100.00	28.57	38.60	175	− 7.72	4.59
Household income group 2	-15.29	-100.00	74.07	29.40	168	–	–
Age 31–45							
Household income group 1	-27.96	-100.00	100.00	41.72	316	− 15.41	3.39
Household income group 2	-12.55	-100.00	100.00	27.72	536	–	–
Age 46–64							
Household Income Group 1	-18.50	-100.00	100.00	36.41	537	− 4.16	2.55
Household income group 2	-14.34	-100.00	100.00	30.22	718	–	–
Age $\ge$ 65							
Household income group 1	-1.57	-100.00	100.00	18.39	55	0.01	2.23
Household income group 2	-1.58	-100.00	100.00	9.84	173	–	–
Panel B. Nov 2020 wave							
Age < 31							
Household income group 1	-7.13	-100.00	100.00	50.60	174	0.11	6.78
Household income group 2	-7.24	-100.00	100.00	44.72	169	–	–
Age 31–45							
Household income group 1	-21.90	-100.00	100.00	40.24	318	− 15.17	3.32
Household income group 2	-6.73	-100.00	100.00	28.90	534	–	–
Age 46–64							
Household income group 1	-13.29	-100.00	100.00	35.09	541	− 1.49	2.64
Household income group 2	-11.80	-100.00	95.24	30.50	714	–	–
Age $\ge$ 65							
Household income group 1	-3.59	-100.00	66.67	30.07	56	0.91	6.69
Household income group 2	-4.50	-100.00	100.00	18.23	172	–	–

Columns 1 to 5 of this table present the statistics used in Fig. 5. Columns 6 and 7 examine whether the change in individual income is statistically different across household income groups. Group 1 corresponds to quintiles 1 and 2. Group 2 corresponds to quintiles 3, 4, and 5. For the subsample defined by each panel, we regress the change in individual income on the indicator for group 1. Column 6 reports the point estimates and column 7 reports the standard errors. The F-statistic of joint significance of household income group 1 and its interactions with the age dummies is 29.03 in panel A and 5.21 in panel B

**Table 9:** Change in individual income, by employment status

	Mean	Min	Max	SD	Count	Diff	se (diff)
	(1)	(2)	(3)	(4)	(5)	(6)	(7)
Panel A. May 2020 wave							
Self-employed							
Household income group 1	-45.69	-100.00	100.00	48.45	74	–15.76	8.25
Household income group 2	-29.93	-100.00	100.00	35.80	126	–	–
Salaried							
Household income group 1	-28.45	-100.00	100.00	39.43	437	–16.43	2.77
Household income group 2	-12.02	-100.00	100.00	27.51	983	–	–
Unemployed							
Household income group 1	-20.37	-100.00	100.00	40.02	298	0.96	4.74
Household income group 2	-21.33	-100.00	40.00	35.01	125	–	–
Retired							
Household income group 1	-2.32	-100.00	100.00	17.21	120	0.82	1.79
Household income group 2	-3.14	-100.00	100.00	14.73	275	–	–
Panel B. Nov 2020 wave							
Self-employed							
Household income group 1	-29.04	-100.00	100.00	43.34	69	–12.52	7.84
Household income group 2	-16.52	-100.00	51.28	25.48	131	–	–
Salaried							
Household income group 1	-15.81	-100.00	100.00	41.35	456	–7.55	2.92
Household income group 2	-8.27	-100.00	100.00	26.24	964	–	–
Unemployed							
Household income group 1	-17.87	-100.00	100.00	44.26	286	0.73	8.33
Household income group 2	-18.59	-100.00	100.00	58.81	137	–	–
Retired							
Household income group 1	-5.02	-100.00	66.67	27.66	125	–1.09	4.15
Household income group 2	-3.93	-100.00	100.00	18.93	270	–	–

Columns 1 to 5 of this table present the statistics used in Figure 6. Columns 6 and 7 examine whether the change in individual income is statistically different across household income groups. Group 1 corresponds to quintiles 1 and 2. Group 2 corresponds to quintiles 3, 4, and 5. For the subsample defined by each panel, we regress the change in individual income on the indicator for group 1. Column 6 reports the point estimates and column 7 reports the standard errors. The F-statistic of joint significance of household income group 1 and its interactions with the employment status dummies is 27.86 in panel A and 5.97 in panel B

**Table 10:** Change in individual income of salaried and self-employed income, by essential sector

	Mean	Min	Max	SD	Count	Diff	se (diff)
	(1)	(2)	(3)	(4)	(5)	(6)	(7)
Panel A. May 2020 wave							
Self-employed & non-essential							
Household income group 1	-56.28	-100.00	100.00	46.94	44	− 21.53	10.36
Household income group 2	-34.75	-100.00	100.00	36.11	90	–	–
Self-employed & Essential							
Household income group 1	-25.27	-100.00	100.00	48.40	24	− 17.55	10.63
Household income group 2	-7.72	-76.19	0.00	17.93	30	–	–
Salaried & Non-Essential							
Household income group 1	-43.38	-100.00	100.00	40.42	232	− 20.94	4.05
Household income group 2	-22.44	-100.00	100.00	33.23	471	–	–
Salaried & essential							
Household income group 1	-12.52	-100.00	95.24	31.11	185	−9.75	3.08
Household income group 2	-2.78	-100.00	100.00	16.56	471	–	–
Panel B. Nov 2020 wave							
Self-employed & Non-Essential							
Household income group 1	-32.36	-100.00	100.00	42.93	44	−12.27	10.39
Household income group 2	-20.09	-100.00	28.57	26.54	90	–	–
Self-employed & essential							
Household income group 1	-26.12	-100.00	28.57	41.17	21	−18.36	10.61
Household income group 2	-7.75	-59.26	51.28	18.24	33	–	–
Salaried & non-essential							
Household income group 1	-23.35	-100.00	100.00	45.34	241	− 10.63	4.45
Household income group 2	-12.73	-100.00	100.00	31.18	462	–	–
Salaried & essential							
Household income group 1	-6.62	-100.00	100.00	34.43	193	− 2.24	3.74
Household income group 2	-4.38	-100.00	95.24	19.70	463	–	–

Columns 1 to 5 of this table present the statistics used in Fig. 7. Columns 6 and 7 examine whether the change in individual income is statistically different across household income groups. Group 1 corresponds to quintiles 1 and 2. Group 2 corresponds to quintiles 3, 4, and 5. For the subsample defined by each panel, we regress the change in individual income on the indicator for group 1. Column 6 reports the point estimates and column 7 reports the standard errors. The F-statistic of joint significance of household income group 1 and its interactions with the essential sector dummies is 34.45 in panel A and 7.27 in panel B

**Table 11:** Change in individual income of salaried employees, by type of contract

	Mean	Min	Max	Sd	Count	Diff	se (diff)
	(1)	(2)	(3)	(4)	(5)	(6)	(7)
Panel A. May 2020 wave							
Temporary							
Household income group 1	-31.27	-100.00	100.00	43.30	256	− 15.36	4.63
Household income group 2	-15.92	-100.00	100.00	34.76	221	–	–
Permanent							
Household income group 1	-20.33	-100.00	95.24	35.30	397	− 8.42	2.55
Household income group 2	-11.91	-100.00	100.00	26.09	857	–	–
Panel B. Nov 2020 wave							
Temporary							
Household income group 1	-19.17	-100.00	100.00	48.50	259	− 4.18	5.95
Household income group 2	-14.99	-100.00	100.00	44.32	218	–	–
Permanent							
Household income group 1	-14.68	-100.00	100.00	35.30	407	− 7.18	2.49
Household income group 2	-7.51	-100.00	100.00	26.13	847	–	–

*Notes:* Columns 1 to 5 of this table present the statistics used in Figure 8. Columns 6 and 7 examine whether the change in individual income is statistically different across household income groups. Group 1 corresponds to quintiles 1 and 2. Group 2 corresponds to quintiles 3, 4, and 5. For the subsample defined by each panel, we regress the change in individual income on the indicator for group 1. Column 6 reports the point estimates and column 7 reports the standard errors. The F-statistic of joint significance of household income group 1 and its interaction with the temporary dummy is 10.78 in panel A and 4.8 in panel B

**Table 12:** Well-being Levels and Change, by Income

	Mean	Min	Max	Sd	Count	Diff	se (diff)
	(1)	(2)	(3)	(4)	(5)	(6)	(7)
*Panel A. May 2020 wave*							
Quintile 1	5.25	0.00	10.00	2.47	541	− 1.03	0.20
Quintile 2	5.67	0.00	10.00	2.38	542	− 0.61	0.19
Quintile 3	5.75	0.00	10.00	2.20	607	− 0.53	0.18
Quintile 4	6.00	0.00	10.00	2.25	610	− 0.28	0.18
Quintile 5	6.28	0.00	10.00	2.15	378	–	–
*Panel B. Nov 2020 wave*							
Quintile 1	5.16	0.00	10.00	2.32	627	−0.53	0.17
Quintile 2	5.05	0.00	10.00	2.33	462	−0.63	0.19
Quintile 3	5.38	0.00	10.00	2.34	592	−0.30	0.19
Quintile 4	5.57	0.00	10.00	2.17	591	−0.11	0.17
Quintile 5	5.68	0.00	10.00	2.13	406	–	–

Columns 1 to 5 of this table present the statistics used in Fig. 9. Columns 6 and 7 examine whether well-being levels are statistically different across income quintiles. We regress the reported well-being on quintile dummies, omitting quintile 5. The regression is estimated on the sample corresponding to each sub-panel. Column 6 reports the point estimates and column 7 reports the standard errors. The F-statistic of joint significance of all quintile dummies is 7.57 in panel A and 4.45 in panel B

**Table 13:** Reasons for Loss of Well-being (May 2020), by Income

	Mean	Min	Max	Sd	Count	Diff	se (diff)
	(1)	(2)	(3)	(4)	(5)	(6)	(7)
*Panel A. Uncertainty about Future*							
Quintile 1	0.85	0.00	1.00	0.36	237	0.03	0.05
Quintile 2	0.83	0.00	1.00	0.37	257	0.02	0.05
Quintile 3	0.84	0.00	1.00	0.37	269	0.02	0.05
Quintile 4	0.78	0.00	1.00	0.42	274	− 0.04	0.05
Quintile 5	0.82	0.00	1.00	0.39	144	–	–
*Panel B. Loss Contact with Family*							
Quintile 1	0.42	0.00	1.00	0.49	237	− 0.17	0.07
Quintile 2	0.50	0.00	1.00	0.50	257	− 0.09	0.07
Quintile 3	0.56	0.00	1.00	0.50	269	− 0.02	0.07
Quintile 4	0.55	0.00	1.00	0.50	274	− 0.04	0.07
Quintile 5	0.59	0.00	1.00	0.49	144	–	–
*Panel C. Loss of Income/Employment*							
Quintile 1	0.36	0.00	1.00	0.48	237	0.14	0.06
Quintile 2	0.22	0.00	1.00	0.42	257	0.00	0.06
Quintile 3	0.25	0.00	1.00	0.43	269	0.03	0.05
Quintile 4	0.21	0.00	1.00	0.41	274	− 0.01	0.05
Quintile 5	0.22	0.00	1.00	0.41	144	–	–
*Panel D. Health*							
Quintile 1	0.16	0.00	1.00	0.36	237	0.02	0.05
Quintile 2	0.14	0.00	1.00	0.35	257	0.01	0.05
Quintile 3	0.10	0.00	1.00	0.31	269	− 0.03	0.04
Quintile 4	0.15	0.00	1.00	0.36	274	0.02	0.05
Quintile 5	0.13	0.00	1.00	0.34	144	–	–
*Panel E. Conciliation*							
Quintile 1	0.10	0.00	1.00	0.29	237	0.04	0.03
Quintile 2	0.08	0.00	1.00	0.27	257	0.02	0.03
Quintile 3	0.12	0.00	1.00	0.32	269	0.06	0.03
Quintile 4	0.06	0.00	1.00	0.23	274	0.00	0.03
Quintile 5	0.06	0.00	1.00	0.23	144	–	–

Columns 1 to 5 of this table present the statistics used in Fig. 10. Columns 6 and 7 examine whether the reason for loss of well-being is statistically different across income quintiles. For each panel, we regress a dummy indicating that reason is mentioned on quintile dummies, omitting quintile 5. Column 6 reports the point estimates and column 7 reports the standard errors. The F-statistic of joint significance of all quintile dummies are: Uncertainty about Future = .83; Loss Contact with Family = 2.37; Loss of Income/Employment = 2.6; Health = .64; Conciliation = 1.57

**Table 14:** Reasons for loss of well-being (November 2020), by income

	Mean	Min	Max	SD	Count	Diff	se (diff)
	(1)	(2)	(3)	(4)	(5)	(6)	(7)
Panel A. Uncertainty about Future							
Quintile 1	0.76	0.00	1.00	0.43	307	-0.01	0.06
Quintile 2	0.81	0.00	1.00	0.39	244	0.03	0.05
Quintile 3	0.79	0.00	1.00	0.41	299	0.01	0.05
Quintile 4	0.74	0.00	1.00	0.44	286	-0.03	0.06
Quintile 5	0.78	0.00	1.00	0.42	176	–	–
Panel B. Loss Contact with Family							
Quintile 1	0.69	0.00	1.00	0.46	307	-0.15	0.05
Quintile 2	0.76	0.00	1.00	0.43	244	-0.08	0.05
Quintile 3	0.70	0.00	1.00	0.46	299	-0.13	0.05
Quintile 4	0.78	0.00	1.00	0.41	286	-0.05	0.04
Quintile 5	0.84	0.00	1.00	0.37	176	–	–
Panel C. Loss of Income/Employment							
Quintile 1	0.37	0.00	1.00	0.48	307	0.20	0.05
Quintile 2	0.33	0.00	1.00	0.47	244	0.17	0.05
Quintile 3	0.31	0.00	1.00	0.46	299	0.15	0.05
Quintile 4	0.24	0.00	1.00	0.43	286	0.08	0.05
Quintile 5	0.16	0.00	1.00	0.37	176	–	–
Panel D. Health							
Quintile 1	0.20	0.00	1.00	0.40	307	0.09	0.04
Quintile 2	0.21	0.00	1.00	0.41	244	0.10	0.04
Quintile 3	0.19	0.00	1.00	0.39	299	0.07	0.04
Quintile 4	0.13	0.00	1.00	0.33	286	0.01	0.04
Quintile 5	0.11	0.00	1.00	0.32	176	–	–
Panel E. Conciliation							
Quintile 1	0.05	0.00	1.00	0.21	307	-0.06	0.03
Quintile 2	0.05	0.00	1.00	0.22	244	-0.05	0.03
Quintile 3	0.11	0.00	1.00	0.31	299	0.00	0.04
Quintile 4	0.03	0.00	1.00	0.18	286	-0.07	0.03
Quintile 5	0.10	0.00	1.00	0.31	176	–	–

*Notes:* Columns 1 to 5 of this table present the statistics used in Figure 10. Columns 6 and 7 examine whether the reason for loss of well-being is statistically different across income quintiles. For each panel, we regress a dummy indicating that reason is mentioned on quintile dummies, omitting quintile 5. Column 6 reports the point estimates and column 7 reports the standard errors. The F-statistic of joint significance of all quintile dummies are: Uncertainty about Future = .58; Loss Contact with Family = 3.26; Loss of Income/Employment = 5.13; Health = 2.31; Conciliation = 3.11

## C First-Wave Questionnaire

Answer options are in *italic*, separated by a semicolon.

1. Please, enter your year of birth.
2. You are a...
  *Man; Woman*
3. The following questionnaire aims to collect information on the effects of Covid-19 on the household economy. Your participation is voluntary, completely anonymous, and you can leave the survey at any time. We will ask you a series of questions about your personal and economic situation. We will also give you information that you may find useful about some recent changes in our society.
  The results of this survey will be used by a team of researchers from the Center for Monetary and Financial Studies and other academic institutions for scientific purposes only.
4. Do you agree to participate?
  *Yes; No*

**Table 15:** Change in Household Income, by Quintile, Gender and Family Status

	Dep Var: Indicator for Losing Job (Unemployed or ERTE)			
	Families with kids		No Families without kids	
	Occupation Controls		Occupation Controls	
	(1)	(2)	(3)	(4)
*Dep Var Mean*	*0.328*	*0.324*	*0.328*	*0.326*
Female	$-0.012$	$-0.239$	0.119	0.175
	(0.100)	(0.239)	(0.114)	(0.253)
High Income	$-0.340$***	$-0.265$***	$-0.210$**	$-0.108$
	(0.080)	(0.080)	(0.085)	(0.093)
Female x High Income	0.102	0.166	$-0.053$	$-0.052$
	(0.107)	(0.111)	(0.120)	(0.125)
Observations	640	636	780	777
R-squared	0.059	0.209	0.029	0.211

Notes: The unit of observation is the individual respondent. The sample is restrited to individuals that were salaried before the pandemic. The dependent variable takes value one if those individuals lost their job with the pandemic (i.e., became unemployed or where under a temporary layoff scheme). High income dummy is an indicator for quintiles 2, 3, 4, and 5. Columns 2 and 4 include controls for types of occupation dummies interacted with a female dummy. Robust standard errors in parenthesis. *** p< 0.01, ** p<0.05, * p<0.1

### C.0.1 Basic demographic information

5. In which autonomous community do you live?
  *Andalucí*a; Aragón; Cantabria; Castilla y León; Castilla-La Mancha; Cataluña; Ceuta; Comunidad de Madrid; Comunidad Foral de Navarra; Comunidad Valenciana; Extremadura; Galicia; Islas Baleares; Islas Canarias; La Rioja; Melilla; País Vasco; Principado de Asturias; Región de Murcia
6. In which province do you live?
  *A Coruñ*a; Alacant/Alicante; Álava; Albacete; Almería; Asturias; Ávila; Badajoz; Barcelona; Burgos; Cáceres; Cádiz; Cantabria; Castelló/Castellón; Ciudad Real; Córdoba; Cuenca; Girona; Granada; Guadalajara; Guipúzcoa; Huelva; Huesca; Islas Baleares; Jaén; La Rioja; Las Palmas; León; Lleida; Lugo; Madrid; Málaga; Murcia; Navarra; Ourense; Palencia; Pontevedra; Salamanca; Santa Cruz de Tenerife; Segovia; Sevilla; Soria; Tarragona; Teruel; Toledo; Valencia; Valladolid; Vizcaya; Zamora; Zaragoza
7. What is the highest educational or work qualification you have?
  *No studies at all; Primary education; First stage of Secondary Education (ESO); Second stage of Secondary Education (Bachillerato); Vocational education (Intermediate Level); Vocational education (Advanced Level); Incomplete university studies; University Studies (Diploma); University Studies (Bachelor’s Degree); Master’s Degree; PhD; Other; I prefer not to answer*
  [If the respondent answers something different to *“Other”*, go to question 9.]
8. Could you please specify what is the highest educational or work qualification you have?
  *Text box*
9. How many people, including yourself, live in your household? Please, indicate the number of people for each of the following age groups:
  - Minors (0 to 17 years old)
  - Adults (18 to 64 years old)
  - Adults (65+ years old)
  *Text box (one for each age group)*
10. What is your nationality?
  *Spanish; Other (please specify)*
  [If the respondent answers *“Spanish”*, go to question 6.]
11. How many years have you been legally resident in Spain, if any?
  *I do not have legal residence; Less than 1 year; 1 to 5 years; More than 5 years; I prefer not to answer*

### C.0.2 Employment situation

**Employment situation before the state of alarm (March 1, 2020)**

12. Which of the following options best represents your employment status before the state of alarm (this is, before March 1, 2020)?
  *Employee (private sector); Employee (public sector); Entrepreneur, professional or self-employed; Retiree or pensioner; Unemployed but I have worked before; Unemployed and I have not worked before; Student; Unpaid domestic work; Other (please specify)*
  [If the respondent answers *“Unemployed but I have worked before”*, go to question 13. If the respondent answers*“Employee (private sector)”, “Employee (public sector)”* or *“Entrepreneur, professional or self-employed”*, go to question 14. Otherwise, go to question 10.]
13. You indicated that you were unemployed before the state of alarm, but that you have worked before. Please, indicate the date on which your last employment contract ended. Please, use the following format: DD/MM/YYYYY (Day/Month/Year). For example: 04/17/2008.
  *Text box*
14. What type of occupation or position best reflects your work activity before the state of alarm (this is, March 1, 2020)?
  *Directors and managers; Professionals, scientists and intellectuals; Technicians and associate professionals; Clerical support workers; Service personnel in the hotel, tourism and catering industry; Service personnel in other sectors; Domestic service; Sales workers; Delivery men and women; Security personnel; Cleaning personnel; Agricultural workers; Officers, and craft and related trades workers; Plant and machine operators, and assemblers; Healthcare personnel (doctors or managers); Healthcare personnel (nurses or assistants); Healthcare personnel (other); Military and police occupations; Other (please specify); I do not know*
15. What type of contract or occupation best reflects your situation before the state of alarm (this is, March 1, 2020)?
  *Salaried employee with indefinite contract (full time); Salaried employee with indefinite contract (part-time); Salaried employee with temporary contract (full time); Salaried employee with temporary contract (part-time); Entrepreneur or professional with employees; Professional or self-employed with no employees; Household chores; Other situation (please specify)*
16. What was the main activity of the company or organization where you were employed before the state of alarm (this is, March 1, 2020)?
  *Agriculture, livestock and primary sector; Extractive industries; Manufacturing industry; Power, gas and water production and distribution; Construction; Retail trade, repair of vehicles and objects; Hotels, tourism, catering; Transportation, warehousing and communications; Financial services; Consulting, advertising or other business services; Real estate activities; Public service; Security and defense services; Education; Health and veterinary services, social services; Culture and sports; Other activities (please specify)*
  [If the respondent answered *“Employee (private sector)”, “Employee (public sector)”* or *“Entrepreneur, professional or self-employed”* to question 6, go to question 17. If the respondent answered *“Unemployed but I have worked before”* to question 6, go to question 10.]

**Employment situation during the state of alarm**

17. Was your job declared “essential” during the state of alarm?
  *Yes; No; I do not know*
18. How did your work situation change as a result of the declaration of the state of alarm on March 14?
  *I continued to work in the same job or occupation; I was included in an ERTE*^33^; *I stopped working when I was laid off or my employment contract ended; I changed my job or started a new job or occupation; Other (please specify)*
  [If the respondent answers *“I continued to work in the same job or occupation”*, go to question 19. If the respondent answers *“I was included in an ERTE*$^{33}$”, go to question 21. If the respondent answers *“I stopped working when I was laid off or my employment contract ended”*, go to question 23. If the respondent answers *“I changed my job or started a new job or occupation”*, go to question 24. If the respondent answers *“Other (please specify)”*, go to question 10.]
19. How has the length of your working day changed?
  *I work as many hours as before; I work fewer hours than before; I work more hours than before*
20. How has the nature of your working day changed?
  *I still work onsite the whole working day; I have switched to teleworking partially; I have switched to teleworking fully; I keep teleworking part or all of my working day, as I already used to do before; Other (please specify)*
  [Go to question 10]
21. Are you still in ERTE^34^ situation?
  *Yes; No*
  [If the respondent answers *“Yes”*, go to question 10.]
22. You indicated that you are no longer in ERTE$^34$ situation. Please, indicate approximately the date in which this situation ended in your job.
  *Text box*
  [Go to question 10.]
23. Have you found a new job?
  *Yes; No*
  [If the respondent answers *“No”*, go to question 10.]

**Employment situation in case of change during the state of alarm**

24. You indicated that you changed your job or started a new job or occupation after the state of alarm was declared. Could you please indicate which of the following options best represents such new job/occupation?
  *Employee (private sector); Employee (public sector); Entrepreneur, professional or self-employed; Retiree or pensioner; Student; Unpaid domestic work; Other (please specify)*
  [If the respondent answers *“Retiree or pensioner”*, *“Student”*, *“Unpaid domestic work”* or *“Other (please specify)”*, go to question 10.]
25. What type of occupation or position best reflects the work activity you started during the state of alarm?
  *Directors and managers; Professionals, scientists and intellectuals; Technicians and associate professionals; Clerical support workers; Service personnel in the hotel, tourism and catering industry; Service personnel in other sectors; Domestic service; Sales workers; Delivery men and women; Security personnel; Cleaning personnel; Agricultural workers; Officers, and craft and related trades workers; Plant and machine operators, and assemblers; Healthcare personnel (doctors or managers); Healthcare personnel (nurses or assistants); Healthcare personnel (other); Military and police occupations; Other (please specify); I do not know*
26. What type of contract or occupation best reflects the new working activity you started during the state of alarm?
  *Salaried employee with indefinite contract (full time); Salaried employee with indefinite contract (part-time); Salaried employee with temporary contract (full time); Salaried employee with temporary contract (part-time); Entrepreneur or professional with employees; Professional or self-employed with no employees; Household chores; Other situation (please specify)*
27. What is the main activity of the company or organization where you started working during the state of alarm?
  *Agriculture, livestock and primary sector; Extractive industries; Manufacturing industry; Power, gas and water production and distribution; Construction; Retail trade, repair of vehicles and objects; Hotels, tourism, catering; Transportation, warehousing and communications; Financial services; Consulting, advertising or other business services; Real estate activities; Public service; Security and defense services; Education; Health and veterinary services, social services; Culture and sports; Other activities (please specify)*
28. Was your job declared “essential” during the state of alarm?
  *Yes; No; I do not know*
29. Has your work situation undergone any change between the last occupation you described and your current situation?
  *Yes; No*
  [If the respondent answers *“No”*, go to question 10.]

**Current employment situation**

30. You indicated that your employment situation changed between the last occupation you described and your current situation. Could you indicate what was this change in your employment situation?
  *I changed my job or started a new job or occupation; I was laid off or my employment contract ended, and now I am unemployed; I was laid off but I have found a new job; I was included in an ERTE*^35^; *Other (please specify)*
  [If the respondent answers *“I was laid off or my employment contract ended, and now I am unemployed”, “I was included in an ERTE*$^35$” or *“Other (please specify)”*, go to question 10.]
31. Which of the following options best represents your current employment status?
  *Employee (private sector); Employee (public sector); Entrepreneur, professional or self-employed; Retiree or pensioner; Student; Unpaid domestic work; Other (please specify)*
  [If the respondent answers *“Retiree or pensioner”*, *“Student”*, *“Unpaid domestic work”* or *“Other (please specify)”*, go to question 10.]
32. What type of occupation or position best reflects your current work activity?
  *Directors and managers; Professionals, scientists and intellectuals; Technicians and associate professionals; Clerical support workers; Service personnel in the hotel, tourism and catering industry; Service personnel in other sectors; Domestic service; Sales workers; Delivery men and women; Security personnel; Cleaning personnel; Agricultural workers; Officers, and craft and related trades workers; Plant and machine operators, and assemblers; Healthcare personnel (doctors or managers); Healthcare personnel (nurses or assistants); Healthcare personnel (other); Military and police occupations; Other (please specify); I do not know*
33. What type of contract or occupation best reflects your current situation?
  *Salaried employee with indefinite contract (full time); Salaried employee with indefinite contract (part-time); Salaried employee with temporary contract (full time); Salaried employee with temporary contract (part-time); Entrepreneur or professional with employees; Professional or self-employed with no employees; Household chores; Other situation (please specify)*
34. What is the main activity of the company or organization where you currently work?
  *Agriculture, livestock and primary sector; Extractive industries; Manufacturing industry; Power, gas and water production and distribution; Construction; Retail trade, repair of vehicles and objects; Hotels, tourism, catering; Transportation, warehousing and communications; Financial services; Consulting, advertising or other business services; Real estate activities; Public service; Security and defense services; Education; Health and veterinary services, social services; Culture and sports; Other activities (please specify)*

#### Individual and household income

35. What was your monthly income, on average, during 2019? Please specify both:
  - Your individual monthly income
  - That of your household as a whole
  By income we mean, for example, wages, income from professional activities, pensions and subsidies, among others. Please indicate the net income, this is, your income after taxes. You do not need to indicate the exact amount, just need to indicate in which interval of the following scale are included your income and the income of your household. We remind you that this information is completely confidential.
  *I have no income at all; 0–300€; 301–600€; 601–900€; 901–1200€; 1201–1500€; 1501–1800€; 1801–2100€; 2101–2400€; 2401–3000€; 3001–4500€; 4501–6000€; 6000€+; I do not know / I prefer not to answer*
36. Thinking about your net monthly income, did it changed during the first weeks of the state of alarm (this is, between March 15 and May 31, 2020)? Please, specify this for:
  - Your individual monthly income
  - That of your household as a whole
  *Yes; No*
37. And, more precisely, how has your net monthly income (after taxes) changed between March 15 and May 31, 2020? Please, specify this for:
  - Your individual monthly income
  - That of your household as a whole
  *Reduced by more than 1000€   per month; Reduced between 600€   and 1000€   per month; Reduced between 400€   and 600€   per month; Reduced between 200 and 400€   euros per month; Reduced between 100 and 200€   euros per month; It is more or less the same; Increased between 100 and 500€  per month; Increased by more than 500€   per month; I do not know; I prefer not to answer*
38. Have you received any public subsidy, aid or benefit during the last 6 months? Check all that apply:
  *Unemployment benefit; ERTE* 36 *benefit; Pension; Moratorium on mortgage or rent payments; Extraordinary benefit for self-employed who cease activity; Other benefits (please specify); I do not know; I have not received any subsidy, aid or public benefit*
39. Has anyone in your household received some Minimum Insertion Income or Guaranteed Income from your autonomous community in the last 6 months?
  *Yes; No; I do not know*
  [If the respondent answers *“No”* or *“I do not know”*, go to question 41.]
40. Please, indicate the approximate monthly amount that your household has received for the Minimum Insertion Income or Guaranteed Income from your autonomous community in the last 6 months.
  *0–99€; 100–199€; 200–299€; 300–399€; 400–499€; 500–599€; 600–699€; 700–799€; 800–899€; 900–1000€; I do not know*

### C.0.4 Videos treatment (only treatment groups)

41. *TREATMENT GROUPS:* The Covid-19 pandemic has greatly changed our lives. Below, we would like to show you some videos that might be of your interest.
  Thank you for your attention!

### C.0.5 Preferences and opinion

42. Next, we want to ask you a series of questions about the economic and political situation of the country.
43. From your point of view, which of the following terms best represents the current situation in Spain? You can select up to two options.
  *Hope; Amelioration; Unity; Solidarity; Uncertainty; Division; Inequality; Deterioration; Despair*

**Trust**

44. Next, we want to ask you about your level of trust regarding a number of entities or groups of people. Using a scale of 0 to 10, where 0 means “I have very little trust in them” and 10 means “I have a lot of trust in them”, how much trust do you have in the following ones?
  - Politicians in the Congress of Deputies
  - Government of Spain
  - Government of your autonomous community
  - Government of your municipality
  - European Union Institutions
  - The judicial system
  - The public health system
  - Epidemiologists
  - Economists
  - Media
  - Pharmaceutical companies
  *0; 1; 2; 3; 4; 5; 6; 7; 8; 9; 10*
45. On a scale of 0 to 10, where 0 is “very low” and 10 is “very high”, how would you rate the ability of the political system to provide solutions to the main problems faced by citizens?
  *0; 1; 2; 3; 4; 5; 6; 7; 8; 9; 10*

**Political preferences**

46. If general elections were to be held again tomorrow, this is, elections to the Spanish Parliament, which party would you vote for?
  *PSOE; PP; Vox; Podemos; Ciudadanos; Má*s País-Equo; ERC-Sobiranistes; Junts-JuntsxCat; CUP-PR; EAJ-PNV; EH Bildu; Other; Abstention/ Would not vote; I do not know
47. Are you one of those people who always votes for the same party, who usually votes for the same party or, depending on what convinces them most at a particular time, votes for one party or another, or does not vote at all?
  *I always vote for the same party; I usually vote for the same party; I vote for one party or another, or I do not vote at all, depending on what convinces me most at a particular time; I tend to vote blank or null; I do not usually vote; I prefer not to answer*
48. When talking about politics, the expressions left and right are commonly used. On a scale of 0 to 10, where 0 means “left” and 10 means “right”, where would you place yourself?
  *0; 1; 2; 3; 4; 5; 6; 7; 8; 9; 10; I prefer not to answer*
49. Please, indicate which party you feel more sympathy for:
  *PSOE; PP; Vox; Podemos; Ciudadanos; Má*s País-Equo; ERC; Junts-JuntsxCat; CUP; EAJ-PNV; EH Bildu; Izquierda Unida; Other; None of them
50. On a scale of 0 to 10, where 0 is “you do not like it” and 10 is “you like it”, how would you rate the following parties?
  - PSOE
  - PP
  - Vox
  - Podemos
  - Ciudadanos
  - ERC
  - EAJ-PNV
  - Más País-Equo
  - CUP
  - Junts-JuntsxCat
  - EH Bildu
  - Izquierda Unida
  *0; 1; 2; 3; 4; 5; 6; 7; 8; 9; 10*
51. On a scale of 0 to 10, where 0 is “very bad” and 10 is “very good”, how would you evaluate the management of the Covid-19 crisis by the Government of Spain?
  *0; 1; 2; 3; 4; 5; 6; 7; 8; 9; 10*

**Participation in collective actions**

52. Since the state of alarm was declared, have you regularly participated in cheering and applause signs of support to the healthcare personnel at 8 pm?
  *Always; Almost always; Few times; Almost never; Never*
53. Since the state of alarm was declared, have you regularly participated in pots and pans protests against the political management?
  *Always; Almost always; Few times; Almost never; Never*
54. Since the state of alarm was declared, have you participated in any protest or demonstration?
  *Yes; No*
  [If the respondent answers* “No”*, go to question 42.]
55. Could you indicate the reason for the protest?
  *Text box*

**Preferences towards redistribution**

56. Please indicate which of the following statements best reflects your opinion about taxes. You can select more than one option if you prefer.
  *Taxes are a means to better redistribute wealth in our society; Taxes are necessary for the state to be able to provide public services; What we pay in taxes does not correspond to the public services we receive, due to corruption; The money that the state collects in taxes would be better off in the pockets of citizens*
57. The Covid-19 crisis has led to an increase in public spending to finance health and social protection measures, such as the ERTE^37^ benefits. Some politicians argue that it is necessary to raise taxes to finance these additional costs, while others propose lowering taxes to revive the economy. Indicate your position on a scale of 0 to 10, where 0 is “lower taxes” and 10 is “raise taxes”.
  *0; 1; 2; 3; 4; 5; 6; 7; 8; 9; 10*
58. If taxes were to be raised, which group do you think should bear the greatest increase in tax payments?
  *Taxpayers with income over 120,000€per year (10,000€per month); Taxpayers with income over 60,000€per year (5000€per month); All taxpayers in proportion to their income*

**Knowledge and opinion about the Minimum Income Scheme **(***Ingreso Mínimo Vital***)

59. On May 29, the government approved the Minimum Vital Income. Do you know this measure?
  *Yes, I know it well; Yes, I know a bit about it; No, I don’t*
60. What is your opinion about the Minimum Vital Income? Please, use the scale of 0 to 10, where 0 means “I do not like it at all” and 10 means “I like it very much”.
  *0; 1; 2; 3; 4; 5; 6; 7; 8; 9; 10*
  [If the respondent answered *“No, I don’t”* to question 82, go to question 62.]
61. Select the option that best captures your opinion about this aid. You can select more than one option if you prefer.
  *It will help to those people most in need; It will help reduce inequality; It will help reduce child poverty; It is too costly for the state; It will discourage beneficiaries to seek employment; It will foster the informal economy; It is not fair to give an aid to people who are able to work; Other (please specify)*
62. Do you know whether your household is entitled to this aid (Minimum Vital Income)?
  *Yes, it is entitled; No, it is not entitled; I do not know*
  [If the respondent answers *“No, it is not entitled”*, go to question 14]
63. Have you (or someone in your household) applied for this benefit or do you intend to apply for it?
  *I have already applied for it; I have not applied for it, but I am going to apply for it; I have not applied for it and I do not know if I will do so; I have not and will not apply for it*
  [If the respondent answers *“I have not and will not apply for it”*, go to question 14.]
64. On a scale of 0 to 10, where 0 is “very difficult” and 10 is “very easy”, how easy do you find the administrative procedures to apply for the aid?
  *0; 1; 2; 3; 4; 5; 6; 7; 8; 9; 10*
65. Which are the main difficulties you find in the administrative procedures to apply for the aid?
  *It requires lots of documents; The process is difficult to understand; The process takes a long time; I do not have a computer; Other (please specify); I do not know*
  [If the respondent answered *“I have already applied for it”* to question 83, go to question 66. If the respondent answered *“I have not applied for it, but I am going to apply for it”* or *“I have not applied for it and I do not know if I will do so”* to question 83, go to question 14.]
66. Have you or anyone in your household received the Minimum Vital Income benefit?
  *Yes; No; I do not know*
67. According to the announcement by the government, the Minimum Vital Income that will be granted to an eligible family consisting of two adults and two children is 877€per month. If this family has an income of 400€per month from their current job, what transfer do you think they should receive?
  *877€per month; 477€per month; 277€per month; No transfer; I do not know*

**Exposure to COVID-19 and health services**

68. In recent months many people have been infected with Covid-19. Below we will ask you some questions about this. Remember that all information you provide will be treated confidentially and used only in aggregate form by our researchers.
69. In the past few months, have you had symptoms consistent with COVID-19?
  *No symptoms; Mild symptoms (for instance, cough or fever less than 38 degrees); Severe symptoms (for instance, fever greater than 38 degrees or breathing difficulties), without hospitalization; Severe symptoms, with hospitalization; I prefer not to answer*
70. Have you been tested for Covid-19? Check all that apply.
  *Yes, PCR test or antigen test with positive result (infected); Yes, PCR or antigen test with negative result (not infected); Yes, antibody test with positive result (past infection); Yes, antibody test with negative result (not infected); No; I prefer not to answer*
71. Has anyone in your household had symptoms consistent with COVID-19? If you live with several people, describe the most severe case.
  *No one in my household has had symptoms; Mild symptoms (for instance, cough or fever less than 38 degrees); Severe symptoms (for instance, fever greater than 38 degrees or breathing difficulties), without hospitalization; Severe symptoms, with hospitalization; Decease; I prefer not to answer*
72. On a scale of 0 to 10, where 0 indicates “great discomfort or depression” and 10 indicates “full happiness”, how would you rate your emotional well-being?
  *0; 1; 2; 3; 4; 5; 6; 7; 8; 9; 10*
73. Comparing your emotional well-being before the state of alarm was declared (March 14, 2020) with your current one, how would you say your emotional well-being has changed?
  *It is now much better; It is now slightly better; About the same; It is now slightly worse; It is now much worse; I prefer not to answer*
  [If the respondent answers *“It is now much better”*, *“It is now slightly better”*, *“About the same”* or *“I prefer not to answer”*, do not answer question 74.]
74. You have previously told us that your emotional well-being has worsened since the state of alarm was declared, select the main reasons. Check all that apply.
  *Loss of employment; Difficulties in reconciling work and child care; Uncertainty about the future; Restrictions on mobility and to go out on the streets; I have reduced my contact with my loved ones; Health problems; Family conflicts; Other (please specify); I prefer not to answer*

## D Second-Wave Questionnaire

Answer options are in *italics*, separated by a semicolon.

1. Last June you responded to a survey on the effects of Covid-19 on the household economy. The following questionnaire is a continuation of the previous one and aims to collect additional information. Thank you for your collaboration!
  Your participation is voluntary, completely anonymous, and you can leave the survey at any time. We will ask you a series of questions about your personal and economic situation. We will also give you information that you may find useful about some recent changes in our society.
  The results of this survey will be used by a team of researchers from the Center for Monetary and Financial Studies and other academic institutions for scientific purposes only.
2. Do you agree to participate?
  *Yes; No*

### D.0.1 Background socio-economic measures: region of residence, level of education, employment situation, sector of occupation, type of contract, current income level, changes in income with respect to before the COVID-19 pandemic

3. In which autonomous community do you live?
  *Andalucí*a; Aragón; Cantabria; Castilla y León; Castilla-La Mancha; Cataluña; Ceuta; Comunidad de Madrid; Comunidad Foral de Navarra; Comunidad Valenciana; Extremadura; Galicia; Islas Baleares; Islas Canarias; La Rioja; Melilla; País Vasco; Principado de Asturias; Región de Murcia
4. What is the highest educational or work qualification you have?
  *Incomplete primary education; Primary education; First stage of Secondary Education (ESO or EGB); Second stage of Secondary Education (Bachillerato, BUP or COU); Vocational education (Intermediate Level); Vocational education (Advanced Level); Incomplete university studies; University Studies (Bachelor’s Degree); Master’s Degree or PhD; Other; I prefer not to answer*
5. What is your age?
6. Which of the following options best represents your current employment status?
  *Employee (private sector); Employee (public sector); Entrepreneur, professional or self-employed; Retiree or pensioner; Unemployed but I have worked before; Unemployed and I have not worked before; Student; Unpaid domestic work; Other (please specify)*;
  [If the respondent answers *“Retiree or pensioner”*, *“Unemployed and I have not worked before”*, *“Student”*, *“Unpaid domestic work”* or *“Other (please specify)”*, go to question 10.]
7. What type of occupation or position best reflects your current work activity?
  *Directors and managers; Professionals, scientists and intellectuals; Technicians and associate professionals; Clerical support workers; Service personnel in the hotel, tourism and catering industry; Service personnel in other sectors; Domestic service; Sales workers; Delivery men and women; Security personnel; Cleaning personnel; Agricultural workers; Officers, and craft and related trades workers; Plant and machine operators, and assemblers; Healthcare personnel (doctors or managers); Healthcare personnel (nurses or assistants); Healthcare personnel (other); Military and police occupations; Teaching personnel; Other (please specify); I do not know*
8. What type of contract or occupation best reflects your current situation?
  *Salaried employee with indefinite contract (full time); Salaried employee with indefinite contract (part-time); Salaried employee with temporary contract (full time); Salaried employee with temporary contract (part-time); Entrepreneur or professional with employees; Professional or self-employed with no employees; Household chores; Other situation (please specify)*
9. What is the main activity of the company or organization where you currently work?
  *Agriculture, livestock and primary sector; Extractive industries; Manufacturing industry; Power, gas and water production and distribution; Construction; Retail trade, repair of vehicles and objects; Hotels, tourism, catering; Transportation, warehousing and communications; Financial services; Consulting, advertising or other business services; Real estate activities; Public service; Security and defense services; Education; Health and veterinary services, social services; Culture and sports; Other personal services; Households employing domestic workers; Activities ancillary to transportation, travel agencies; Computer activities; Other activities (please specify)*
10. What was your monthly income, on average, during 2019? Please specify both:
  - Your individual monthly income
  - That of your household as a whole
  By income we mean, for example, wages, income from professional activities, pensions and subsidies, among others. Please indicate the net income, this is, your income after taxes. You do not need to indicate the exact amount, just need to indicate in which interval of the following scale are included your income and the income of your household. We remind you that this information is completely confidential.
  *I have no income at all; 0–300€; 301–600€; 601–900€; 901–1200€; 1201–1500€; 1501–1800€; 1801–2100€; 2101–2400€; 2401–3000€; 3001–4500€; 4501–6000€; 6000€+; I do not know / I prefer not to answer*
11. Thinking about your net monthly income, has it changed since the Covid-19 epidemic broke out (this is, between February 2020 and now)? Please, specify this for:
  - Your individual monthly income
  - That of your household as a whole
  *Yes; No*
12. And, more precisely, how has your net monthly income (after taxes) changed between February 2020 and today? Please, specify this for:
  - Your individual monthly income
  - That of your household as a whole
  *Reduced by more than 1000€per month; Reduced between 600€and 1000€per month; Reduced between 400€and 600€per month; Reduced between 200€and 400€euros per month; Reduced between 100€and 200€euros per month; It is more or less the same; Increased between 100€and 500€per month; Increased by more than 500€per month; I do not know; I prefer not to answer*
13. Have you received any public subsidy, aid or benefit during the last 6 months? Check all that apply:
  *Unemployment benefit; ERTE* 38 *benefit; Pension; Moratorium on mortgage or rent payments; Minimum Vital Income from the state government; Minimum Insertion Income or Guaranteed Income from your autonomous community; Extraordinary benefit for self-employed who cease activity; Other benefits (please specify); I have not received any subsidy, aid or public benefit; I do not know*
  [If the respondent answers *“Minimum Vital Income from the state government”*, go to question 14. If the respondent answers *“Minimum Insertion Income or Guaranteed Income from your autonomous community”*, go to question 15. Otherwise, go to question 16.]
14. Please, indicate the approximate monthly amount that your household has received or been granted for the Minimum Vital Income in the last few months.
  *0–99€; 100–199€; 200–299€; 300–399€; 400–499€; 500–599€; 600–699€; 700–799€; 800–899€; 900–1000€; 1000–1100€; I do not know*
  [Go to question 16]
15. Please, indicate the approximate monthly amount that your household has received or been granted for the Minimum Insertion Income or Guaranteed Income from your autonomous community in the last few months.
  *0–99€; 100–199€; 200–299€; 300–399€; 400–499€; 500–599€; 600–699€; 700–799€; 800–899€; 900–1000€; 1000–1100€; I do not know*
16. We are interested in knowing the size of the municipality in which you live. Consider very large (more than 1,000,000 people), large (between 500,000 and 1,000,000 people), medium (between 100,000 and 500,000 people), small (between 10,000 and 100,000 people) and very small (less than 10,000 people). To show that you have read the question, select the two answers “Very large” and “Very small” regardless of the reality. What is the size of the municipality in which you have your normal residence?
  *Very small; Small; Medium; Large; Very large*

### D.0.2 Elicitation of priors and treatments (only treatment groups)

17. *TREATMENT GROUPS 1 AND 2:* The Covid-19 pandemic has greatly changed our lives. Below, we would like to show you some information that might be of your interest.
  In the last weeks, harsh measures have been imposed to contain the advance of Covid-19: curfews, mobility restrictions, maximum of 6 people in social gatherings, cancellation of cultural events.
  How did we get here? Could these measures have been avoided with a more efficient management of the pandemic by our governments?
  In March 2020, the scientific community recommended developing mass testing and contact tracing systems. Investing in these systems reduces the spread of the virus and helps to avoid having to take harsher measures.
  Have our politicians done their “homework”? Next, we will give you information about the quality of the tracing system in your autonomous community (at the end of the survey we will give you more information about the data used).
  Do you know how many contact tracers per 100,000 inhabitants there were in your autonomous community in October 2020? Before giving you the exact number, we ask you to try to guess based on the information provided. The colors indicate the following:
  - Red: Very few contact tracers. More than half of cases left un-traced.
  - Orange/Yellow: Insufficient contact tracers. All cases cannot be traced.
  - Green: Adequate number of contact tracers. All cases can be traced.
  *Slider going from 0 to 200*
18. *TREATMENT GROUPS 1 AND 2:* On a scale of 0 to 10, where 0 is “very unsure” and 10 is “very sure”, how sure are you that you were close to the correct number of tracers?
  *0; 1; 2; 3; 4; 5; 6; 7; 8; 9; 10*
19. *TREATMENT GROUPS 1 AND 2:* Your autonomous community has *x* contact tracers per 100,000 inhabitants. With *x* contact tracers, your autonomous community lacks *t-x* contact tracers per 100,000 inhabitants to be able to track all cases.^39^ Deficiencies in tracing contribute to the increase in cases and lead to the application of tougher measures, such as those we have been experiencing in recent weeks.
  [If the respondent answered *“Galicia”* to question 3, display the following paragraph]
  The official number of contact tracers in Galicia includes primary healthcare personnel. In our estimation of the number of contact tracers we have taken into account that primary healthcare personnel only spend part of their working day on tracing tasks. More information is provided at the end of the survey.
20. *TREATMENT GROUP 2:* All the autonomous communities have a lack of contact tracers, but there are big differences across them. How does contact tracing work in your autonomous community compared with other communities in Spain? Next, we give you information about it.
  Your autonomous community is the *y* th worse in terms of contact tracers.

### D.0.3 Assessment of competence, degree of responsibility and trust in different levels of government

21. On a scale of 0 to 10, where 0 is “very bad” and 10 is “very good”, how would you evaluate the management capacity of the government of your autonomous community in dealing with a crisis like the Covid-19 one?
  *0; 1; 2; 3; 4; 5; 6; 7; 8; 9; 10*
22. On a scale of 0 to 10, where 0 is “very bad” and 10 is “very good”, how would you evaluate the management capacity of the Government of Spain in dealing with a crisis like the Covid-19 one?
  *0; 1; 2; 3; 4; 5; 6; 7; 8; 9; 10*
23. We would like to ask you about which institution you think bears greater responsibility in the management of the Covid-19 pandemic in your autonomous community (containment measures, healthcare, contact tracing, testing, etc.).
  On a scale of -10 to 10, where -10 is “all responsibility lies with the Government of Spain” and 10 “all responsibility lies with the government of your autonomous community”, which degree of responsibility would you attribute to each government?
  *-10; -9; -8; -7; -6; -5; -4; -3; -2; -1; 0; 1; 2; 3; 4; 5; 6; 7; 8; 9; 10*
24. We would like to ask you about what factors you think have the most influence on the evolution of the pandemic (this is, on the number of Covid-19 infections). What do you think is more important, government management (containment measures, contact tracing, testing, etc.) or other factors (population density, aging population, etc.)? On a scale of -10 to 10, where -10 is “pandemic depends only on other factors” and 10 is “pandemic depends only on government management”, where would you place yourself?
  *-10; -9; -8; -7; -6; -5; -4; -3; -2; -1; 0; 1; 2; 3; 4; 5; 6; 7; 8; 9; 10*
25. When you consider which party to vote for in general elections, do you take more into account the competence in the management of each party, or the proximity of the party to your ideals? On a scale of -10 to 10, where -10 is “the management of each party” and 10 is “the party’s proximity to your ideals”, where would you place yourself?
  *-10; -9; -8; -7; -6; -5; -4; -3; -2; -1; 0; 1; 2; 3; 4; 5; 6; 7; 8; 9; 10*
26. On a scale of 0 to 10, how would you rate the ability of each of the following parties to manage a crisis similar to the one generated by Covid-19?
  - PP (Partido Popular)
  - PSOE (Partido Socialista Obrero Español)
  - Cs (Ciudadanos)
  - VOX
  - Podemos
  *0; 1; 2; 3; 4; 5; 6; 7; 8; 9; 10*
27. Which level of government do you think has the greatest capacity to manage a crisis similar to the one generated by Covid-19? On a scale of -10 to 10, where -10 is “the government of the autonomous community” and 10 is “the Government of Spain”, where would you place yourself?
  *-10; -9; -8; -7; -6; -5; -4; -3; -2; -1; 0; 1; 2; 3; 4; 5; 6; 7; 8; 9; 10*
28. Next, we want to ask you about your level of trust regarding a number of entities or groups of people. Using a scale of 0 to 10, where 0 means “I have very little trust in them” and 10 means “I have a lot of trust in them”, how much trust do you have in the following ones?
  - Politicians in the Congress of Deputies
  - Government of Spain
  - Government of your autonomous community
  - Government of your municipality
  - European Union Institutions
  - The judicial system
  - The public health system
  - Epidemiologists
  - Economists
  - Media
  - Pharmaceutical companies
  *0; 1; 2; 3; 4; 5; 6; 7; 8; 9; 10*
29. On a scale of 0 to 10, where 0 is “very low” and 10 is “very high”, how would you rate the ability of the political system to provide solutions to the main problems faced by citizens?
  *0; 1; 2; 3; 4; 5; 6; 7; 8; 9; 10*
30. Imagine that you win a prize of 1000€ aimed to alleviate the effects of Covid-19 in the Spanish society. You cannot keep the prize. You can only donate it to the following two institutions. Which percentage of the prize would you donate to each of them?
  - Covid-19 Fund of the Ministry of Health, Government of Spain
  - Red Cross
  *Text box (one for each institution)*
31. Imagine that you win another similar prize of 1000€  and the two institutions to which you can donate it are the following ones. Which percentage of the prize would you donate to each of them?
  - Fund against Covid-19 of the Health Department of your autonomous community
  - Red Cross
  *Text box (one for each institution)*

### D.0.4 Voting Intentions and sympathies towards political parties

32. From your point of view, which of the following terms best represents the current situation in Spain? You can select up to two options.
  *Hope; Amelioration; Unity; Solidarity; Uncertainty; Division; Inequality; Deterioration; Despair*
33. If a general election were to be held tomorrow, this is, an election to the Spanish Parliament, which party would you vote for?
  *PSOE; PP; Vox; Unidas Podemos; Ciudadanos; Má*s País-Equo; ERC-Sobiranistes; Junts-JuntsxCat; CUP-PR; EAJ-PNV; EH Bildu; Other (please specify); Abstention/ Would not vote; I do not know
34. If regional elections were to be held again tomorrow, this is, elections to the Parliament of your Autonomous Community, which party would you vote for?
  *List of political parties adapted to the answer to question 3.*
35. Are you one of those people who always votes for the same party, who usually votes for the same party or, depending on what convinces them most at a particular time, votes for one party or another, or does not vote at all?
  *I always vote for the same party; I usually vote for the same party; I vote for one party or another, or I do not vote at all, depending on what convinces me most at a particular time; I tend to vote blank or null; I do not usually vote; I prefer not to answer*
36. When talking about politics, the expressions left and right are commonly used. On a scale of 0 to 10, where 0 means “left” and 10 means “right”, where would you place yourself?
  *0; 1; 2; 3; 4; 5; 6; 7; 8; 9; 10; I prefer not to answer*
37. Please, indicate which party you feel more sympathy for:
  *PSOE; PP; Vox; Podemos; Ciudadanos; Má*s País-Equo; ERC; Junts-JuntsxCat; CUP; EAJ-PNV; EH Bildu; Izquierda Unida; Other (please specify); None of them
38. On a scale of 0 to 10, where 0 is “you do not like it” and 10 is “you like it”, how would you rate the following parties?
  - PSOE
  - PP
  - Vox
  - Podemos
  - Ciudadanos
  - ERC
  - EAJ-PNV
  - Más País-Equo
  - CUP
  - Junts-JuntsxCat
  - EH Bildu
  - Izquierda Unida
  *0; 1; 2; 3; 4; 5; 6; 7; 8; 9; 10*
39. Since the Covid-19 epidemic broke out (this is, February 2020), have you regularly participated in pots and pans protests against the political management?
  *Always; Almost always; Few times; Almost never; Never*
40. Since the Covid-19 epidemic broke out (this is, February 2020), have you participated in any protest or demonstration?
  *Yes; No*
  [If the respondent answers*“No”*, go to question 42.]
41. Could you indicate the reason for the protest?
  *Text box*

### D.0.5 Preferences towards redistribution

42. Please indicate which of the following statements best reflects your opinion about taxes. You can select more than one option if you prefer.
  *Taxes are a means to better redistribute wealth in our society; Taxes are necessary for the state to be able to provide public services; What we pay in taxes does not correspond to the public services we receive, due to corruption; The money that the state collects in taxes would be better off in the pockets of citizens*
43. The Covid-19 crisis has led to an increase in public spending to finance health and social protection measures, such as the ERTE^40^ benefits. Some politicians argue that it is necessary to raise taxes to finance these additional costs, while others propose lowering taxes to revive the economy. Indicate your position on a scale of 0 to 10, where 0 is “lower taxes” and 10 is “raise taxes”.
  *0; 1; 2; 3; 4; 5; 6; 7; 8; 9; 10*
44. If taxes were to be raised, which group do you think should bear the greatest increase in tax payments?
  *Taxpayers with income over 120,000€per year (10,000€per month); Taxpayers with income over 60,000€per year (5000€per month); All taxpayers in proportion to their income*

### D.0.6 Compliance with COVID-19 prevention directives

45. In recent months many people have been infected with Covid-19. Below we will ask you some questions regarding potential containment measures. Remember that all information you provide will be treated confidentially and used only in aggregate form by our researchers.
46. The government of your autonomous community recommends people to wear masks, also outdoors, even if a safety distance of 2 meters can be maintained. Please indicate which of the following statements best reflects your opinion about this measure. Check all that apply.
  *I think it is a good measure. It is important to wear a mask to protect everyone’s health; It seems excessive to me. It should only be mandatory indoors, and outdoors when a distance of 2 meters cannot be maintained; It seems excessive to me. It should only be mandatory indoors; The use of masks should not be mandatory. It is an imposition against individual freedom; Other (please specify)*
47. The government of your autonomous community requires people who have been in close contact with a person infected with Covid-19 to be confined to home for at least 10 days. These are called “quarantines”. If you were in such a situation, would you comply with this indication? We remind you that your answer is completely confidential.
  *Yes, I would stay at home for 10 days or more; I would try to leave my house as least as possible for 10 days; It would be impossible for me not to leave home for 10 days due to professional and/or family responsibilities; I would not follow such directions. I would act according to my own judgment; Other (please specify); I prefer not to answer*
48. Based on what you see in your neighborhood or municipality, which do you think is the degree of compliance with quarantines and other restrictions by people similar to you? Please, indicate on a scale of 0 to 10, where 0 is “rarely met” and 10 is “strictly met”.
  *0; 1; 2; 3; 4; 5; 6; 7; 8; 9; 10*
49. Imagine that in the next few months a vaccine against Covid-19 is approved. Imagine that the Government of Spain recommends vaccination in your age group. How likely would you be to follow the government’s recommendation and agree to be vaccinated?
  *I would certainly accept to be vaccinated; It is likely that I would accept to be vaccinated; I do not know whether or not I would accept to be vaccinated; It is likely that I would not accept to be vaccinated; I would certainly not accept to be vaccinated; I do not know*
50. If, instead, the government of your autonomous community was to recommend vaccination in your age group, how likely would you be to follow this recommendation and agree to be vaccinated?
  *I would certainly accept to be vaccinated; It is likely that I would accept to be vaccinated; I do not know whether or not I would accept to be vaccinated; It is likely that I would not accept to be vaccinated; I would certainly not accept to be vaccinated; I do not know*

### D.0.7 Open ended questions on the economic situation and management of the COVID-19 pandemic

51. The Covid-19 crisis has greatly changed our lives. We are really interested in your views on how the situation has been handled. Below we ask you some questions and leave some boxes for you to tell us your vision. You can write as much as you like. We will be happy to read it. Thank you very much.
52. When you think about the economic situation, what aspects seem most relevant to you?
  *Text box*
53. What is your opinion on how the Covid-19 pandemic has been managed in Spain?
  *Text box*
54. When you think about the impact of Covid-19 and its impact on the economy, which population groups are you most concerned about?
  *Text box*

### D.0.8 Usefulness of treatment information (only treatment groups)

55. *TREATMENT GROUPS 1 AND 2:* On a scale of 0 to 10, where 0 is “not useful at all” and 10 is “very useful”, how useful did you find the information on the number of contact tracers in your autonomous community that we have provided in this survey?
  *0; 1; 2; 3; 4; 5; 6; 7; 8; 9; 10*
  [If the respondent answers *“5”* or greater, go to question 63.]
56. *TREATMENT GROUPS 1 AND 2:* Your answer above indicates that the information provided on the number of contact tracers has not been very useful to you. Could you tell us the reason(s)? Please, check all that apply.
  *I already knew this information; I do not consider this information relevant; I have doubts about the quality of the data provided; I do not agree with some of the statements; Other (please specify)*

### D.0.9 Elicitation of priors and usefulness of treatment information (only control group)

57. *CONTROL GROUP:* The Covid-19 pandemic has greatly changed our lives. Below, we would like to show you some information that might be of your interest.
  In the last weeks, harsh measures have been imposed to contain the advance of Covid-19: curfews, mobility restrictions, maximum of 6 people in social gatherings, cancellation of cultural events.
  How did we get here? Could these measures have been avoided with a more efficient management of the pandemic by our governments?
  In March 2020, the scientific community recommended developing mass testing and contact tracing systems. Investing in these systems reduces the spread of the virus and helps to avoid having to take harsher measures.
  Have our politicians done their homework? Next, we will give you information about the quality of the tracing system in your autonomous community (at the end of the survey we will give you more information about the data used).
  Do you know how many contact tracers per 100,000 inhabitants there were in your autonomous community in October 2020? Before giving you the exact number, we ask you to try to guess based on the information provided. The colors indicate the following:
  - Red: Very few contact tracers. More than half of cases left un-traced.
  - Orange/Yellow: Insufficient contact tracers. All cases cannot be traced.
  - Green: Adequate number of contact tracers. All cases can be traced.
  *Slider going from 0 to 200*
58. *CONTROL GROUP:* On a scale of 0 to 10, where 0 is “very unsure” and 10 is “very sure”, how sure are you that you were close to the correct number of tracers?
  *0; 1; 2; 3; 4; 5; 6; 7; 8; 9; 10*
59. *CONTROL GROUP:* Your autonomous community has *x* contact tracers per 100,000 inhabitants. With *x* contact tracers, your autonomous community lacks *t-x* contact tracers per 100,000 inhabitants to be able to track all cases.^41^ Deficiencies in tracing contribute to the increase in cases and lead to the application of tougher measures, such as those we have been experiencing in recent weeks.
  [If the respondent answered *“Galicia”* to question 3, display the following paragraph]
  The official number of contact tracers in Galicia includes primary healthcare personnel. In our estimation of the number of contact tracers we have taken into account that primary healthcare personnel only spend part of their working day on tracing tasks. More information is provided at the end of the survey.
60. *CONTROL GROUP:* All the autonomous communities have a lack of contact tracers, but there are big differences across them. How does contact tracing work in your autonomous community compared with other communities in Spain? Next, we give you information about it.
  Your autonomous community is the *y* th worse in terms of contact tracers.
61. *CONTROL GROUP:* On a scale of 0 to 10, where 0 is “not useful at all” and 10 is “very useful”, how useful did you find the information on the number of contact tracers in your autonomous community that we have provided in this survey?
  *0; 1; 2; 3; 4; 5; 6; 7; 8; 9; 10*
  [If the respondent answers *“5”* or greater, go to question 63.]
62. *CONTROL GROUP:* Your answer above indicates that the information provided on the number of contact tracers has not been very useful to you. Could you tell us the reason(s)? Please, check all that apply.
  *I already knew this information; I do not consider this information relevant; I have doubts about the quality of the data provided; I do not agree with some of the statements; Other (please specify)*

### D.0.10 Exposure to COVID-19 and health services

63. Below, we would like to ask you some questions about how you have felt over the last few months. We remind you that your answers are completely confidential.
64. In the past few months, have you had symptoms consistent with COVID-19?
  *No symptoms; Mild symptoms (for instance, cough or fever less than 38 degrees); Severe symptoms (for instance, fever greater than 38 degrees or breathing difficulties), without hospitalization; Severe symptoms, with hospitalization; I prefer not to answer*
65. Have you been tested for Covid-19? Check all that apply.
  *Yes, PCR test or antigen test with positive result (infected); Yes, PCR or antigen test with negative result (not infected); Yes, antibody test with positive result (past infection); Yes, antibody test with negative result (not infected); No; I prefer not to answer*
  [If the respondent answers *“No”* or *“I prefer not to answer”*, go to question 72]
66. How long did it take from the time you were tested until you received the results?
  *Less than 24h; Between 24 and 48h; 3 to 5 days; 5 to 10 days; More than 10 days; I did not receive the results; I do not know, I prefer not to answer*
  [If the respondent answered *“Yes, PCR test or antigen test with positive result (infected)”* or *“Yes, antibody test with positive result (past infection)”* to question 65, go to question 67. If the respondent answered *“Yes, PCR or antigen test with negative result (not infected)”* or *“Yes, antibody test with negative result (not infected)”* to question 65, go to question 72.]
67. After your positive Covid-19 test result, did healthcare personnel contact you to follow up on your health status?
  *Yes; No*
  [If the respondent answers “No”, go to question 69]
68. How many times did healthcare personnel contact you during the 14 days following your positive Covid-19 test result?
  *0 (never); 1; 2; 3; 4; 5; 6; 7; 8; 9; 10*
69. After your positive Covid-19 test result, were you contacted for an interview to gather information about the people you had been in contact with in previous days?
  *Yes; No*
  [If the respondent answers *“No”*, go to 72]
70. Do you know whether the contact tracing system, after the interview, reached out to any of the people you had been in contact with?
  *Yes, all of them were contacted; Yes, most of them were contacted; Yes, they contacted some of them; I am not aware that they were contacted; I do not know*
  [If the respondent answers *“I am not aware that they were contacted”* or *“I do not know”*, go to question 72]
71. Approximately, how long did it take for the contact tracing system to phone the people you had been in contact with?
  *They called them within the first 24 hours after my positive test result; They called them within 24 to 48 hours after my positive test result; They called them within 48 to 72 hours after my positive test result; They called them more than 72 hours after my positive test result*
72. Have you been reached out, at any time, by personnel from the contact tracing system to alert you that you may had been in contact with a person who had tested positive for Covid-19?
  *Yes; No; I do not know; I prefer not to answer*
72. During the last few months, have you had difficulties accessing health services in general (for instance, due to cancelled or delayed medical appointments)?
  *Yes; No; I prefer not to answer*
74. We would like to know your views on the risk of Covid-19 infection in your municipality. How likely do you think it is that you will be infected with Covid-19 in the following month?
  *Extremely likely, Very likely; Somewhat likely; Unlikely; Very Unlikely*
75. And how likely do you think it is that a person in his/her 30s, who works on site and lives in your neighborhood or municipality will be infected with Covid-19 in the following month?
  *Extremely likely, Very likely; Somewhat likely; Unlikely; Very Unlikely*
76. How many of your personal acquaintances have been infected with Covid-19?
  *0; 1; 2; 3 to 5; 6 to 10; 10+*
77. On a scale of 0 to 10, where 0 is “very bad” and 10 is “very good”, how would you rate the quality of care received by you and those close to you in the following areas?
  - Covid-19 detection system (speed of tests, results notification, etc.)
  - Contact tracing system
  - Follow-up healthcare for Covid-19 cases
  - Access to the healthcare system (telephone accessibility, availability of appointments, etc.)
  - Medical attention by healthcare personnel
  *0; 1; 2; 3; 4; 5; 6; 7; 8; 9; 10*
78. We would like to know how your habits have changed as a result of Covid-19. Comparing your habits before the start of the pandemic with your current habits, how has the frequency with which you perform the following actions changed?
  - Eating or drinking in restaurants or bars (indoors)
  - Eating or drinking in restaurants or bars (outdoors/terrace)
  - Meeting with close relatives who do not live with you (parents, siblings, aunts and uncles, nieces and nephews).
  - Meeting with other, more distant, relatives
  - Meeting with friends
  - Leaving my municipality for leisure purposes (travel, visits, etc.)
  *Much more frequently; More frequently; As before; Less frequently; Much less frequently*

### D.0.11 Emotional Well-Being

79. On a scale of 0 to 10, where 0 indicates “great discomfort or depression” and 10 indicates “full happiness”, how would you rate your emotional well-being?
  *0; 1; 2; 3; 4; 5; 6; 7; 8; 9; 10*
80. Comparing your emotional well-being before the first state of alarm was declared (March 14, 2020) with your current one, how would you say your emotional well-being has changed?
  *It is now much better; It is now slightly better; About the same; It is now slightly worse; It is now much worse; I prefer not to answer*
  [If the respondent answers *“It is now much better”*, *“It is now slightly better”*, *“About the same”* or *“I prefer not to answer”*, go to question 74.]
81. You have previously told us that your emotional well-being has worsened since the state of alarm was declared, select the main reasons. Check all that apply.
  *Loss of employment and/or income; Difficulties in reconciling work and child care; Uncertainty about the future; I have reduced my contact with my loved ones; Health problems; Family conflicts; Other (please specify); I prefer not to answer*

### D.0.12 Knowledge and opinion about the Minimum Income Scheme (*Ingreso Mí*nimo Vital)

82. On May 29, the government approved the Minimum Vital Income, which aims to guarantee a minimum income for all families. Have you (or someone in your household) applied for this benefit or do you intend to apply for it?
  *I have already applied for it; I have not applied for it, but I am going to apply for it; I have not applied for it and I do not know if I will do so; I have not and will not apply for it*
  [If the respondent answers something different to *“I have already applied for it”*, go to question 85.]
83. Have you received a response to your application for the Minimum Vital Income benefit?
  *Yes, we have been granted the aid and we have received it; Yes, we have been granted the aid but we have not yet received it; Yes, the aid has been denied; We have no response yet*
  [If the respondent answers *“Yes, the aid has been denied”* or *“We have no response yet”*, go to question 85.]
84. Please, indicate the approximate monthly amount that your household has been granted as Minimum Vital Income.
  *0€-99€; 100€-199€; 200€-299€; 300€-399€; 400€-499€; 500€-599€; 600€-699€; 700-799€; 800€-899€; 900€-1000€; 1,000€-1,100€; I do not know*
85. What is your opinion about the Minimum Vital Income? Please, use the scale of 0 to 10, where 0 means “I do not like it at all” and 10 means “I like it very much”.
  *0; 1; 2; 3; 4; 5; 6; 7; 8; 9; 10*
86. Select the option that best captures your opinion about this aid. You can select more than one option if you prefer.
  *It will help to those people most in need; It will help reduce inequality; It will help reduce child poverty; It is too costly for the state; It will discourage beneficiaries to seek employment; It will foster the informal economy; It is not fair to give an aid to people who are able to work; Other (please specify); I do not know, I prefer not to answer*
87. According to the announcement by the government, the Minimum Vital Income that will be granted to an eligible family consisting of two adults and two children is 877€per month. If this family has an income of 400€per month from their current job, what transfer do you think they should receive?
  *877€per month; 477€per month; 277€per month; No transfer; I do not know*
88. Do you want to tell us something more? In the space below you can give us your opinion on this survey or on any of the topics covered, we will be happy to read it!
  *Text box*

### D.0.13 Additional information on sources of information used in the information treatment

89. Additional Information
  - There is extensive scientific evidence on the importance of contact tracing.
  - Here^42^ you can find an scholarly article published in the prestigious journal The Lancet (in English).
  - The calculation of the number of contact tracers needed has been done using the U.S. Health Resources and Services Administration (HRSA) tool.
    The calculation takes into account the population of each autonomous community and the number of cases per 100,000 inhabitants. Here^43^ you can find the tool (in English).
  - The estimated number of contact tracers per inhabitant in each autonomous community has been calculated using data supplied by the regional Health Departments, and information provided by Elena G. Sevillano and Pablo Linde.
    Here^44^ you can find more information.
  [If the respondent answered *“Galicia”* to question 3, display the following paragraph]
  - As we said, there is no reliable data for Galicia, because the autonomous community also considers personnel who are not exclusively dedicated to this task. You can find more information here.^45^
    For the calculation of the number of contact tracers needed to track all cases, we have assumed that the staff spends 10% of their working day on this task.

## Footnotes

1 https://doi.org/10.2907/EUSILC2004-2019V.2.

2 Note that in terms of labor market outcomes, the Spanish Labor Force Survey (EPA) is timely published quarterly. These data provide information on a large sample of individuals representative at the national level. However, they do not contain overall earnings, income, or information on household characteristics or economic situation. Hence, it is not well suited to provide a comprehensive measure of income inequality for the Spanish population.

3 *Financial Times*., Covid: why Spain is hit worse than the rest of Europe, Sept 2020.

4 The survey question referred to total income. Hence, it may include earnings from labor and capital earnings, as well as government transfers and other types of income. See Sect. 2 for details.

5 The magnitudes of the earning losses in the USA and the UK are similar to the ones we estimate for Spain, ranging from around 30% for poorer individuals to around 10% for the more affluent. The drop is around 1/3 smaller in Germany across the whole distribution. Two caveats to interpret these difference are that Adams-Prassl et al. (2020) focus on individuals that had done some paid work in the past 12 months, while we study the whole adult population and that their outcomes are measured in March, while ours are in May (and November).

6 For the case of Spain the estimated Gini coefficient takes values of 0.339 in January 2020, 0.345 in May 2020, and 0.323 in September 2020.

7 GDP per capita dropped by 5.9% between the fourth trimester of 2019 and the second trimester of 2020, and the unemployment rate grew from 13.78 to 15.33% in this period. The change in GDP between the last trimester of 2019 and the last trimester of 2020 was $-9.9$%. Source: INE. Source 1, Source 2.

8 The field work for the first wave started in June and lasted until early July 2020. 93% of respondents submitted the questionnaire in June and 7% of them in July. The field work for the second wave started in late-November and finished during December. 16% of respondents submitted the questionnaire in November and 84% in December.

9 https://es.yougov.com.

10 CIVIO - Description of Essential Activities during the State of Alarm.

11 COVID-19 Status Report 123, June 2020, Spanish Health Ministry.

12 COVID-19 Status Report 261, November 2020, Spanish Health Ministry.

13 Only the measures of well-being were collected after the experimental part. However, the results presented in this paper are similar if we control for the effects of the experiments. The experiment in the first wave provided information treatments about the severity of the economic crisis and the policy response to a randomly selected treatment group. In the second wave, we provided information on the management of the COVID response across different regions.

14 This is achieved through a quota-sampling system. The population is first segmented into mutually exclusive subgroups of age, gender, region, and education level. Then, individuals are contacted from Yougov’s panel of respondents until all quotas are filled.

15 The first wave of our study sampled 5051 individuals that were all recontacted during the second wave. Of them, 3297 completed the questionnaire in the second wave. From this sample we dropped 619 individuals because they did not report complete information on income, occupation, or well-being.

16 These regions correspond to Nielsen areas, which are frequently used by data analytics firms.

17 In Appendix Table 3, we evaluate how well our sample matches inequality measures and, more generally, the income distribution of the Spanish population according to EU-SILC, the European armonized household survey. The Spain this survey is also known as Living Conditions Survey (*Encuesta de Condiciones de Vida*). For the year 2019, the Gini coefficient measured in EU-SILC is 33, while it is 36 in our sample. The income deciles are similarly distributed, albeit our measured incomes are lower in levels relative to the data from EU-SILC.

18 Specifically, weights are computed by Yougov using the random iterative method, calibrated to the marginal distributions of age, gender, region, and education level of the population according to 2019 National Institute of Statistics (INE) data.

19 We infer years of education from education categories. We used the following correspondence to years of education: primary schooling or less = 5, secondary education = 10, upper secondary (ESO) = 12, vocational training (FP) =10, higher education = 16.

20 Note that our measure of income may include both labor and capital income. In particular, earnings from professional activities may include earnings from self employment or from business profits. However, returns from capital are likely to represent a small fraction of household total income, except for those in the very top income deciles.

21 We defined narrow intervals in order to have high precision in their self-reported income. The intervals offered are the following (all expressed in €   per month): 0, 0–300, 300–600, 601–900, 901–1200, 1201–1500, 1501–1800, 1801–2100, 2101–2300, 2401–3000, 3001–4500, 4501–6000, more than 6000. We consider 8000€   as the proxy of income for the highest interval. Few individuals select the largest income bracket. Hence, the precise income estimate for the top income bracket will not have a large effect on our results.

22 The intervals provided were the following (all expressed in €   per month): reduced by more than 1000, reduced between 600 and 1000, reduced between 400 and 600, reduced between 200 and 400, reduced between 100 and 200, stayed the same, increased by 100–500, increased by more than 500.

23 In some instances the reported 2019 income-level differs between the first and second wave. This is natural since we did not ask for income levels for a specific month of 2019, but for the “average month”. To calculate the percent income changes, we divide the reported change in income by the level of income in 2019, as reported in the same survey. This is the most natural option, since respondents probably had in mind the reported 2019 income level when answered to the *change* in incomes between the start of the pandemic and the current moment. Furthermore, we winsorize changes in income by replacing income changes higher than 100% by that number. This imputation is done for 53 observations.

24 *Source*: INE. National Income Statistics.

25 In the first wave, respondents could also choose mobility restrictions, which were not an issue during the second wave. 50% of respondents selected this option.

26 In Appendix Fig.  11, we represent the Lorenz curve and its evolution during this period.

27 Source: World Bank Data.

28 See Appendix Table 4 for additional measures of inequality estimated using our data.

29 See, however, Bell et al. (2021) for an study that finds greater income losses for men than for women.

30 Note that including additional dummies for the other income quintiles does not change the main take-away of the results and makes the presentation slightly more cumbersome.

31 Note that individuals that were unemployed shortly before the pandemic may have been employed during 2019. Hence, there are multiple reasons why they may have experienced changes in income, from changes in labor income, changes in their unemployment subsidies, or changes in the earnings from the informal economy. Our income question was comprehensive by design in order to incorporate multiple sources of income and provide a good proxy for cash-at-hand of individuals.

32 See Sect.  2 for a description of “essential” economic activities and its implications.
